# Endothelial glycocalyx injury in bacterial bloodstream infection: biological determinants and association with host response aberrations

**DOI:** 10.3389/fcimb.2026.1821582

**Published:** 2026-06-02

**Authors:** Hui Wang, Joe M. Butler, Erik H. A. Michels, Tom D. Y. Reijnders, Tjitske S. R. van Engelen, Alex F. de Vos, Olaf L. Cremer, Hessel Peters-Sengers, Tom van der Poll

**Affiliations:** 1 Amsterdam University Medical Center (UMC), Location University of Amsterdam, Center for Infection and Molecular Medicine (CIMM), and Amsterdam Institute for Immunology and Infectious Diseases (AI&I), Amsterdam, Netherlands; 2 Department of Intensive Care Medicine, University Medical Center (UMC) Utrecht, Utrecht, Netherlands; 3 Department of Epidemiology and Data Science, Amsterdam University Medical Center (UMC), Location Free University, Amsterdam, Netherlands; 4 Division of Infectious Diseases, Amsterdam University Medical Center (UMC), Location University of Amsterdam, Amsterdam, Netherlands

**Keywords:** biomarkers, bloodstream infection, endothelium, glycocalyx, sepsis, syndecan-1

## Abstract

**Introduction:**

Damage to the endothelial glycocalyx plays a key role in the pathogenesis of sepsis. Syndecan-1 is a widely used biomarker of glycocalyx degradation. The objectives of this study were (1) to determine whether the pathogen type is correlated with differences in glycocalyx disruption in critically ill patients with bacterial bloodstream infection (BSI) and (2) to assess the association of plasma syndecan-1 levels with host response changes implicated in the pathogenesis of sepsis.

**Methods:**

We measured 24 plasma biomarkers reflective of key pathophysiological domains and whole-blood transcriptomes in intensive care unit (ICU) patients with bacterial BSI. Plasma syndecan-1 was used as biomarker of glycocalyx degradation.

**Results:**

In 188 ICU patients, the most common pathogens were *Escherichia coli* (n =38), *Streptococcus* spp. (n =34), *Enterococcus* spp. (n =25), and *Staphylococcus aureus* (n =23). Higher syndecan-1 was independently associated with increased 30-day mortality. Disease severity explained most syndecan-1 variance (19.2%), whereas pathogen category (6.7%) and infection site (2.2%) contributed little. High syndecan-1 was associated with host response changes, particularly those related to endothelial dysfunction, followed by inflammation and coagulation. Restricted cubic spline regression, mapping biomarker variation across the range of syndecan-1 levels, suggested that some host response anomalies were most pronounced at relatively high degree of glycocalyx degradation (impairment of endothelial barrier function, signs of consumptive coagulopathy), whereas others showed stronger associations at lower syndecan-1 levels (endothelial cell activation and injury). Analysis of the blood transcriptome in a subgroup of patients (n =142) revealed enhanced expression of hemostasis-related pathways, notably platelet degranulation, in patients with elevated syndecan-1 levels.

**Discussion:**

In ICU patients with BSI, glycocalyx disruption as measured by plasma syndecan-1 is associated with poor clinical outcomes and a variety of host response aberrations. Glycocalyx disruption in BSI appears to relate more strongly to disease severity than to the causative pathogen category.

## Introduction

Sepsis causes more than 11 million deaths worldwide each year (Rudd et al., 2020). Sepsis is driven by a dysregulated host response to infection, with strong interactions between systemic inflammation, coagulation activation, and endothelial cell activation and dysfunction (Singer et al., 2016; Wiersinga and van der Poll, 2022; Maneta et al., 2023; Girardis et al., 2024). Together, they shape the pathophysiological mechanisms underlying sepsis (Singer et al., 2016; Wiersinga and van der Poll, 2022; Maneta et al., 2023; Girardis et al., 2024).

Activation of endothelial cells plays a key role in the host response during sepsis (Girardis et al., 2024; van der Poll and Parker, 2020). The surface of endothelial cells is covered by the glycocalyx, a dense layer of polymers made up of proteoglycans, glycosaminoglycans, glycoproteins, and glycolipids (Uchimido et al., 2019; Iba and Levy, 2019; Moore et al., 2021; Iba et al., 2024). In normal physiology, the glycocalyx helps maintain the vascular barrier, supports interactions between cells, provides an anticoagulant surface, and keeps blood flow conditions stable (Uchimido et al., 2019; Iba and Levy, 2019; Moore et al., 2021; Iba et al., 2024). During sepsis, the structural integrity of the glycocalyx is compromised, resulting in fluid extravasation, immune cell adhesion, platelet aggregation and intensified tissue injury. When the glycocalyx is degraded, many of its components are released into the bloodstream, with the proteoglycan syndecan-1 being one of the primary constituents (Iba and Levy, 2019; Hahn et al., 2021). The plasma concentrations of syndecan-1 are commonly used to assess glycocalyx degradation *in vivo* (Uchimido et al., 2019; Fernández-Sarmiento et al., 2023; Sun et al., 2022; Saoraya et al., 2021). In sepsis, higher plasma syndecan-1 levels have been linked to fluid overload, need for dialysis, and greater risk of death (Fernández-Sarmiento et al., 2023; Sun et al., 2022). Compared to other biomarkers of glycocalyx degradation, such as endocan and hyaluronic acid, syndecan-1 shows better performance in predicting the risk of these complications (Fernández-Sarmiento et al., 2023; Becker et al., 2015). Animal studies showed that stopping syndecan-1 shedding can protect the glycocalyx, reduce organ damage, and improve survival, pointing to its potential as a treatment target (Cui et al., 2015; Iba et al., 2020; de Oliveira and Miranda, 2024).

Disruption of the glycocalyx in sepsis involves multiple mechanisms, including direct enzymatic attack, inflammatory and oxidative stress, acute and chronic comorbidities, and hemodynamic instability (Uchimido et al., 2019; Iba and Levy, 2019; Iba et al., 2024). Knowledge of the biological drivers of endothelial glycocalyx breakdown in sepsis *in vivo* is limited. One factor could be the causative pathogen. Indeed, bacteria can cause endothelial injury through a variety of mechanisms (Park et al., 2001, Park et al., 2004; Lubkin and Torres, 2017; Obino and Duménil, 2019) and sepsis patients with positive blood cultures (BCs) show evidence of more severe glycocalyx degradation than BC negative sepsis patients (Hippensteel et al., 2019). We argued that if the causative pathogen plays a role in glycocalyx degradation during sepsis, this effect would be most evident in patients with positive BCs, as the presence of the pathogen in the bloodstream brings it into direct contact with the vascular endothelium. Thus, our first objective was to evaluate the association between the causative pathogen and other factors with glycocalyx disruption in patients admitted to the intensive care unit (ICU) with microbiologically confirmed bacterial bloodstream infection (BSI). In addition, considering the broad influence of the glycocalyx on inflammatory and procoagulant responses (Uchimido et al., 2019; Iba and Levy, 2019; Iba et al., 2024), our second objective was to obtain insight in the involvement of glycocalyx degradation in dysregulation of other pathways implicated in sepsis pathogenesis.

## Materials and methods

### Study design and population

We used the Molecular Diagnosis and Risk Stratification of Sepsis (MARS, 2013-2018) prospective cohort of two mixed ICUs in tertiary teaching hospitals (Academic Medical Center in Amsterdam and University Medical Center Utrecht) (van Vught et al., 2016; Butler et al., 2025; Peters-Sengers et al., 2022). We identified patients with a BC taken between one day before and one day after ICU admission and selected those with a positive BC result for bacteria (Butler et al., 2025). For all bacteremic patients, the source of infection was identified as described (Peters-Sengers et al., 2022). BSIs were classified as mixed when more than one pathogen was detected within a two-day window. Non-infectious ICU controls were defined as described (Butler et al., 2025). From this cohort, a subset of patients also had a PAXgene tube (Becton-Dickinson) collected for transcriptomic analysis between one day before and one day after the BC draw. For definitions of comorbidities, acute respiratory distress syndrome (ARDS) and acute kidney injury (AKI) see the **Supplementary Materials**.

### Biomarker assays

EDTA plasma samples were collected at ICU admission; plasma was obtained from left-over samples drawn for routine patient care by the laboratories of Amsterdam UMC/AMC and UMC Utrecht. Plasma was isolated in a single centrifugation step according to local routine laboratory procedures. Twenty-four biomarkers were measured (**Supplementary Materials**, **Supplementary Table 1**), of which 22 were measured using the Luminex 200 (R&D Systems, Minneapolis, MN) and BioPlex 200 system (BioRad, Hercules, CA). Prothrombin time and platelet counts were extracted from routine clinical records within ± one day of the BC draw. This biomarker panel was selected to characterize major host-response domains implicated in sepsis, rather than to directly assess syndecan-1 shedding pathways. Based on literature, biomarkers were categorized according to three pathophysiological domains (Maneta et al., 2023; Girardis et al., 2024; Pierrakos et al., 2020; Curtiaud et al., 2025).

### Whole-blood transcriptomic analyses

Blood samples for RNA analysis were collected using PaxGene tubes (Becton Dickinson). Whole-blood transcriptomics were generated using RNA sequencing, Affymetrix U219 microarrays, and Thermo Fisher’s GeneChip Human Transcriptome Array (HTA) 2.0. The Coconut R package was applied for correction to harmonize data across batches and platforms (Sweeney, 2017). For details see (Butler et al., 2025) and the Supplementary Materials.

### Statistical analysis

**Figure 1:**
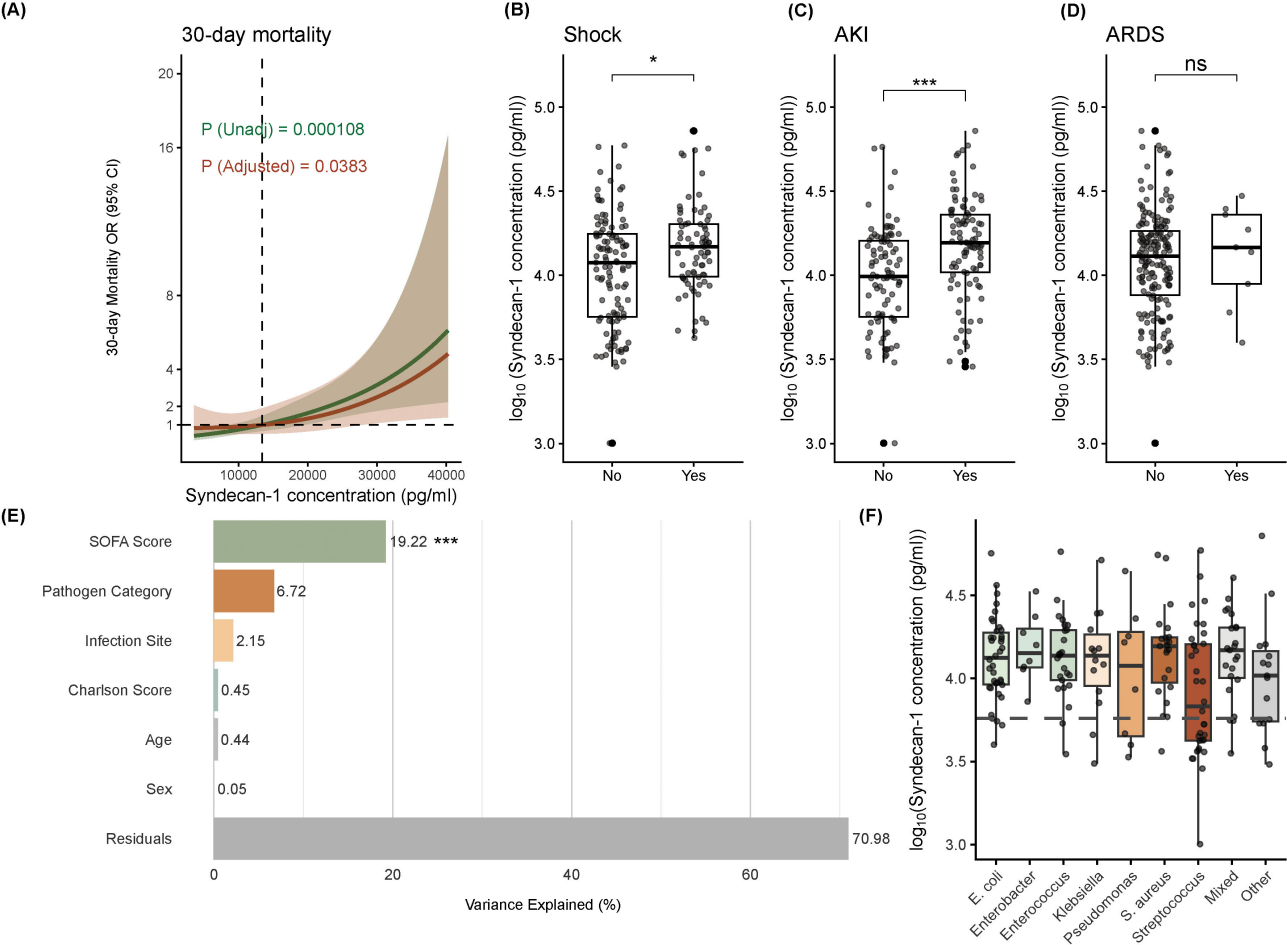
Plasma syndecan-1 in critically ill patients with bacterial bloodstream infection: association with mortality and organ dysfunction, and factors contributing to its variation. **(A)** Spline model of syndecan-1 and 30-day mortality risk. Given the nonlinear relationship between syndecan-1 and mortality, a restricted cubic spline logistic regression model with three inner knots was used to estimate the 30-day mortality risk. The green curve shows the unadjusted model; the brown curve shows the model adjusted for age, gender, chronic kidney disease, cerebrovascular disease, prior myocardial infarction, diabetes and total SOFA score. Odds ratios (OR) were calculated relative to the cohort median (vertical dashed line); an OR > 1 indicates a higher risk with increasing syndecan-1 levels. Shaded areas represent 95% confidence intervals. **(B-D)** Box plots showing syndecan-1 levels (log_10_-transformed) in patients with and without organ dysfunction within the first 24 hours of ICU admission, with each dot representing a patient. **(E)** Contribution of BSI pathogens and other variables to variation in plasma syndecan-1 levels. Linear regression on log_10_-transformed syndecan-1 levels, with the following predictions: SOFA score, pathogen type, infection site, comorbidities (chronic kidney disease, cerebrovascular disease, prior myocardial infarction, diabetes, immunosuppression), age and sex. Bars represent the proportion of total variance in log_10_-transformed Syndecan-1 levels explained by each covariate in the linear regression model. Residuals are what is left. Colors reflect variable categories. **(F)** Box plots of log_10_-transformed syndecan-1 levels by pathogen group, with each dot representing a patient. Colors indicate the pathogen. Group differences were tested using the Kruskal-Wallis test with Dunn’s correction. The dashed line represents the median syndecan-1 concentration in non-infected ICU controls who were initially suspected of infection but in whom all cultures remained negative. AKI, acute kidney injury; ARDS, adult respiratory distress syndrome; SOFA, sequential organ failure assessment; *P. aeruginosa, Pseudomonas aeruginosa; S. aureus*, *Staphylococcus aureus.* *P < 0.05; ***P < 0.001; ns, non-significant.

No formal sample size calculation was conducted considering this was an exploratory hypothesis-generating study. All analyses were done in R (version 4.4.1). Syndecan-1 was analyzed as a continuous variable and by tertile grouping. Biomarker values were log_10_-transformed to reduce skewness and stabilize the variance. Distribution was checked using histograms and Q-Q plots. For skewed variables, we used the Kruskal-Wallis test with Dunn’s *post hoc* test; for normally distributed variables, we used one-way analysis of variance (ANOVA) with Tukey’s test. To assess the association between syndecan-1 levels and 30-day mortality, we built logistic regression models in which syndecan-1 was modeled using restricted cubic splines (3 knots). Non-linearity was evaluated using the overall Wald χ² test from the anova() function in the rms package. Models were run unadjusted and adjusted for age, sex, chronic kidney disease, stroke, prior myocardial infarction, diabetes, and sequential organ failure assessment (SOFA) score. For tabular reporting, odds ratios and 95% confidence intervals were derived at the 75th and 90th percentiles of syndecan-1, using the cohort median as reference; the same median reference was used in **Figure 1A**. We also compared survival across syndecan-1 tertiles using Kaplan-Meier curves and log-rank tests. We fitted a multivariable linear regression model with log-transformed syndecan-1 as the dependent variable, including SOFA score, pathogen type, infection site, demographics and Charlson score as predictors. We then used ANOVA to decompose the model sum of squares and expressed the proportion of explained variance attributable to each predictor. As a sensitivity analysis, this model was repeated with log-transformed plasma creatinine added as a renal function marker, while total SOFA was replaced by non-renal SOFA.

We analyzed host response markers using Principal Component Analysis (PCA). Input markers came from endothelial cell (dys)function, systemic inflammation and coagulation activation. Pearson’s r was used to assess the correlation with the first two components. To quantify differences in biomarker levels between the highest and lowest syndecan-1 tertiles, we used Hedges’ g, a commonly used effect size measure (Hedges, 1981). To examine how individual host response biomarkers vary across the full range of syndecan-1 levels, we first standardized each marker using Z-scores. We then applied restricted cubic spline regression with three knots to model their associations with syndecan-1 and calculated the first derivative to visualize the direction and magnitude of change.

Transcriptomic data were processed with the limma package and empirical Bayes moderation (Ritchie et al., 2015). For gene set enrichment analyses (GSEA), syndecan-1 was analyzed both as a continuous variable and by comparing the highest versus lowest tertiles. We focused on pathways biologically linked to syndecan-1 in the Reactome database: “Hemostasis” (R-HSA-109582) and “Extracellular Matrix Organization” (R-HSA-1474244) (Milacic et al., 2024). All p-values were adjusted using the Benjamini-Hochberg method. Missing values (listed in the **Supplementary Materials**) were assumed to be missing at random and imputed using the mice package in “R” with the CART method (Van Buuren and Groothuis-Oudshoorn, 2011).

## Results

### Baseline characteristics, mortality and organ dysfunction

We enrolled 188 critically ill patients with bacterial BSIs (**Table 1**). The leading pathogens identified from BCs were *Escherichia coli* (n = 38), *Streptococcus* spp. (n = 34), *Enterococcus* spp. (n = 25), and *Staphylococcus aureus* (n = 23). **Supplementary Table 2** lists all cultured BSI pathogens. Plasma syndecan-1 levels in ICU patients with BSI (median 13128.7 pg/ml; IQR 7672.9, 18963.3 pg/ml) were significantly higher than those in non-infectious ICU controls (**Supplementary Table 3**; median 5750.7 pg/ml; IQR 3719.0 – 9366.4 pg/ml; p <0.001). In a logistic regression model plasma syndecan-1 (as a continuous variable) had a significant positive association with 30-day mortality (unadjusted p < 0.001; **Figure 1A**); this relationship remained robust after adjustment for age, sex, key comorbidities, and SOFA score (adjusted p < 0.05). **Supplementary Table 4** provides numerical odds ratios from this model; compared with the cohort median syndecan-1 concentration, the adjusted odds ratios were 1.29 (95% CI 1.00–1.65) at the 75th percentile and 2.19 (95% CI 1.20–4.00) at the 90th percentile. Patients who had shock (n = 75 vs n = 113 without shock) or AKI (n = 95 vs n = 93 without AKI) within 24 hours of ICU admission had higher syndecan-1 levels compared to those without these complications (**Figures 1B,C**). No apparent difference was observed in patients with or without ARDS (n = 9 vs n = 179 without ARDS) (**Figure 1D**).

**Table 1:** Baseline characteristics and clinical outcomes of critically ill patients with bloodstream infection.

n	188
Syndecan-1 (pg/ml), median [IQR]	13128.7 [7672.9, 18963.3]
Demographics	
Age, years, median [IQR]	63 [53, 71]
Sex, male, n (%)	119 (63.3)
Body Mass Index, median [IQR]	24.49 [22.3, 27.2]
Comorbidities	
Chronic obstructive pulmonary disease, n (%)	21 (11.2)
Congestive heart failure, n (%)	7 (3.7)
Prior myocardial infarction, n (%)	17 (9.0)
Cerebrovascular disease, n (%)	16 (8.5)
(Prior) malignancy, n (%)	53 (28.2)
Immunosuppression, n (%)	38 (20.2)
Chronic kidney disease, n (%)	34 (18.1)
Diabetes, n (%)	37 (19.7)
Severity on admission	
APACHE IV, median [IQR]	84.50 [63.8, 108.0]
SOFA score, median [IQR]	8.00 [7.0, 11.0]
Shock, n (%)	75 (39.9)
Acute kidney injury, n (%)	95 (50.5)
Acute respiratory distress syndrome, n (%)	9 (4.8)
Routine laboratory markers	
Platelet counts (x10^9^/L), median [IQR]	140 [64, 226]
Leukocyte counts (x10^9^/L), median [IQR]	14.7 [7.6, 21.8]
Creatinine (µmol/L), median [IQR]	145 [102, 212]
Bilirubin (µmol/L), median [IQR]	18 [9, 44]
Pathogen group*	
*Escherichia coli*, n (%)	38 (20.2)
*Enterobacter*, n (%)	8 (4.3)
*Enterococcus species*, n (%)	25 (13.3)
*Klebsiella species*, n (%)	14 (7.4)
*Pseudomonas aeruginosa*, n (%)	8 (4.3)
*Staphylococcus aureus*, n (%)	23 (12.2)
*Streptococcus species*, n (%)	34 (18.1)
Other, n (%)	15 (8.0)
Mixed, n (%)	23 (12.2)
Source of infection^#^	
Abdominal, n (%)	51 (27.1)
Cardiovascular, n (%)	16 (8.5)
Central nervous system, n (%)	7 (3.7)
Respiratory, n (%)	32 (17.0)
Skin, n (%)	26 (13.8)
Urinary, n (%)	30 (16.0)
Other, n (%)	17 (9.0)
Unknown, n (%)	9 (4.8)
Clinical course	
Length of hospital stay, days, median [IQR]	16.1 [7.8, 42.2]
ICU mortality, n (%)	47 (25.0)
30-day mortality, n (%)	61 (32.4)
90-day mortality, n (%)	80 (42.6)

*For detailed overview of pathogens see **Supplementary Table 2**. APACHE, acute physiology and chronic health evaluation. #Patients can have multiple sources of infection, and percentages represent the number of each source out of the BSI group sizes. SOFA: sequential organ failure assessment. Continuous data are displayed as median [interquartile range] and compared using Kruskal–Wallis test; categorical data are displayed as count (percentage) and compared using Fisher’s exact test.

We next stratified BSI patients into syndecan-1 tertiles to explore trends across different concentrations (**Supplementary Table 5**). Disease severity (SOFA and APACHE IV scores) was significantly higher in patients with higher syndecan-1 levels, while the distribution of BSI pathogens was not different between syndecan-1 tertiles (p = 0.221). The proportion of patients with shock was higher in the middle and highest tertiles, whereas the proportion with AKI within 24 hours of ICU admission increased across tertiles. Mortality was highest among patients with highest syndecan-1 tertiles; **Supplementary Figure 1** shows a Kaplan-Meier plot of 30-day mortality (log-rank p < 0.001).

These results are in agreement with previous reports in patients with sepsis (Fernández-Sarmiento et al., 2023) and highlight a consistent association between elevated syndecan-1 and adverse outcomes in ICU patients with BSI.

### Factors associated with syndecan-1 elevation

We next examined how different clinical and infection-related factors relate to variability in plasma syndecan-1 concentrations. Disease severity showed the strongest association, with SOFA score accounting for 19.2% of the variation in syndecan-1 levels. Pathogen category and infection site contributed more modestly (6.7% and 2.2%, respectively), whereas Charlson score, age, and sex explained little additional variation (**Figure 1E**). In a sensitivity analysis adding log-transformed plasma creatinine to this model, the SOFA score remained the largest measured contributor, while plasma creatinine explained 3.1% of syndecan-1 variance and the contribution of the pathogen category remained modest (6.2%) (**Supplementary Figure 2**).

Since different bacteria can cause various levels of endothelial injury (Park et al., 2001, Park et al., 2004; Lubkin and Torres, 2017; Obino and Duménil, 2019), we analyzed syndecan-1 levels across BSI pathogen groups (**Figure 1F**). Differences between pathogen groups were not significant (p = 0.157).

### Association of syndecan-1 with other host response changes

**Figure 2:**
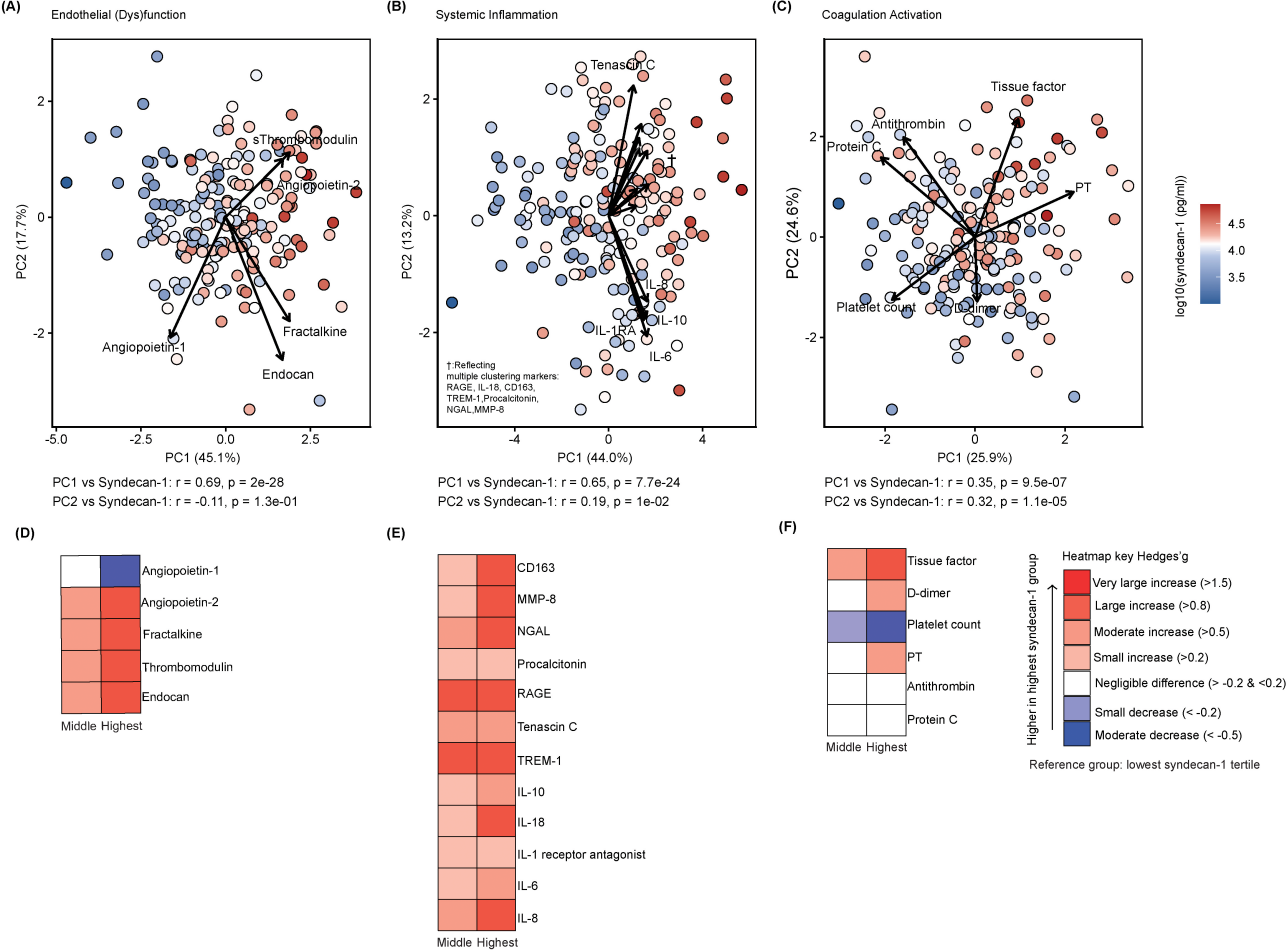
Association of syndecan-1 with host response biomarkers stratified according to pathophysiological domains. **(A-C)** Principal component analysis (PCA) was performed on three groups of host response biomarkers, corresponding to **(A)** Endothelial cell (dys)function. **(B)** Systemic inflammation. **(C)** Coagulation activation. Syndecan-1 was treated as a continuous variable and correlated with principal component (PC)1 and PC2 using Pearson’s correlation coefficient (r). Each point represents one patient, colored according to log_10_-transformed syndecan-1 levels. The x- and y-axes show PC1 and PC2 respectively, with the percentage of variance explained in parentheses. Black arrows indicate the direction and contribution of each biomarker to the PCs; longer arrows represent a more substantial influence. **(D-F)** Association of syndecan-1 with individual host response biomarkers stratified by syndecan-1 tertiles. The heatmaps show Hedges’ g effect sizes for each marker. The lowest tertile of syndecan-1 was used as the reference. The left column compares the middle tertile to the lowest; the right column compares the highest to the lowest. Red indicates higher marker levels in higher syndecan-1 groups. Blue indicates lower levels. Color intensity reflects the strength of the difference. Markers are grouped by biological function domain: **(D)** Endothelial cell (dys)function. **(E)** Systemic inflammation. **(F)** Coagulation activation. CD, cluster of differentiation; IL, interleukin; MMP, matrix metalloproteinase; NGAL, neutrophil gelatinase-associated lipocalin; PT, prothrombin time; RAGE, receptor for advanced glycation end products; TREM, triggering receptor expressed on myeloid cells.

To investigate the association between glycocalyx degradation, as indicated by elevated syndecan-1 levels, and other host response aberrations we analyzed 24 biomarkers reflective of three key pathophysiological domains: endothelial (dys)function, systemic inflammation and coagulation activation (**Supplementary Table 1**) (Girardis et al., 2024; van der Poll and Parker, 2020; Pierrakos et al., 2020; Curtiaud et al., 2025). We first performed PCA to assess the overall association between syndecan-1 (as a continuous variable and by tertile groups) and biomarkers from each domain (**Figures 2A–C**; **Supplementary Figure 3**). Biomarker loadings for PC1 and PC2 are shown in **Supplementary Table 6**. The strongest correlation was observed with PC1 of the endothelial (dys)function domain (r = 0.69), which was primarily driven by angiopoietin-2, fractalkine, and soluble thrombomodulin (**Figure 2A**; **Supplementary Figure 3A**). Syndecan-1 also showed a strong positive correlation with PC1 of the systemic inflammation domain (r = 0.65), which was mainly influenced by neutrophil gelatinase-associated lipocalin (NGAL), soluble triggering receptor expressed by myeloid cells (TREM)-1, and interleukin (IL)-6 (**Figure 2B**; **Supplementary Figure 3B**). In contrast, the correlation with PC1 of the coagulation activation domain was weaker (r = 0.35), with prothrombin time, Protein C, and platelet count contributing most to this component (**Figure 2C**; **Supplementary Figure 2C**). Considering that antithrombin and protein C levels were missing in 19.7% of patients, we performed a sensitivity analysis for the coagulation domain PCA including only patients in whom all biomarkers were measured (n = 151); this analysis yielded similar results (**Supplementary Figure 4**). These results suggest that, among patients with BSI, syndecan-1 elevation is most closely linked to endothelial dysfunction, followed by systemic inflammation, with coagulation disturbances playing a lesser role.

To further evaluate the association between syndecan-1 concentrations and individual host response markers, we determined differences in biomarker levels across syndecan-1 tertiles. **Figures 2D–F** shows the effect sizes (Hedges’ g) (Hedges, 1981) for differences in biomarker concentrations compared with those in the lowest syndecan-1 tertile (used as reference). In agreement with the PCA, the plasma levels of most biomarkers were higher in the middle and upper syndecan-1 tertiles compared to the lowest, particularly those associated with endothelial activation and inflammation; angiopoietin-1 and platelet counts declined with increasing syndecan-1, while changes in antithrombin and protein C were minimal. **Supplementary Table 7** lists absolute biomarker concentrations in BSI patients and stratified according to syndecan-1 tertiles.

**Figure 3:**
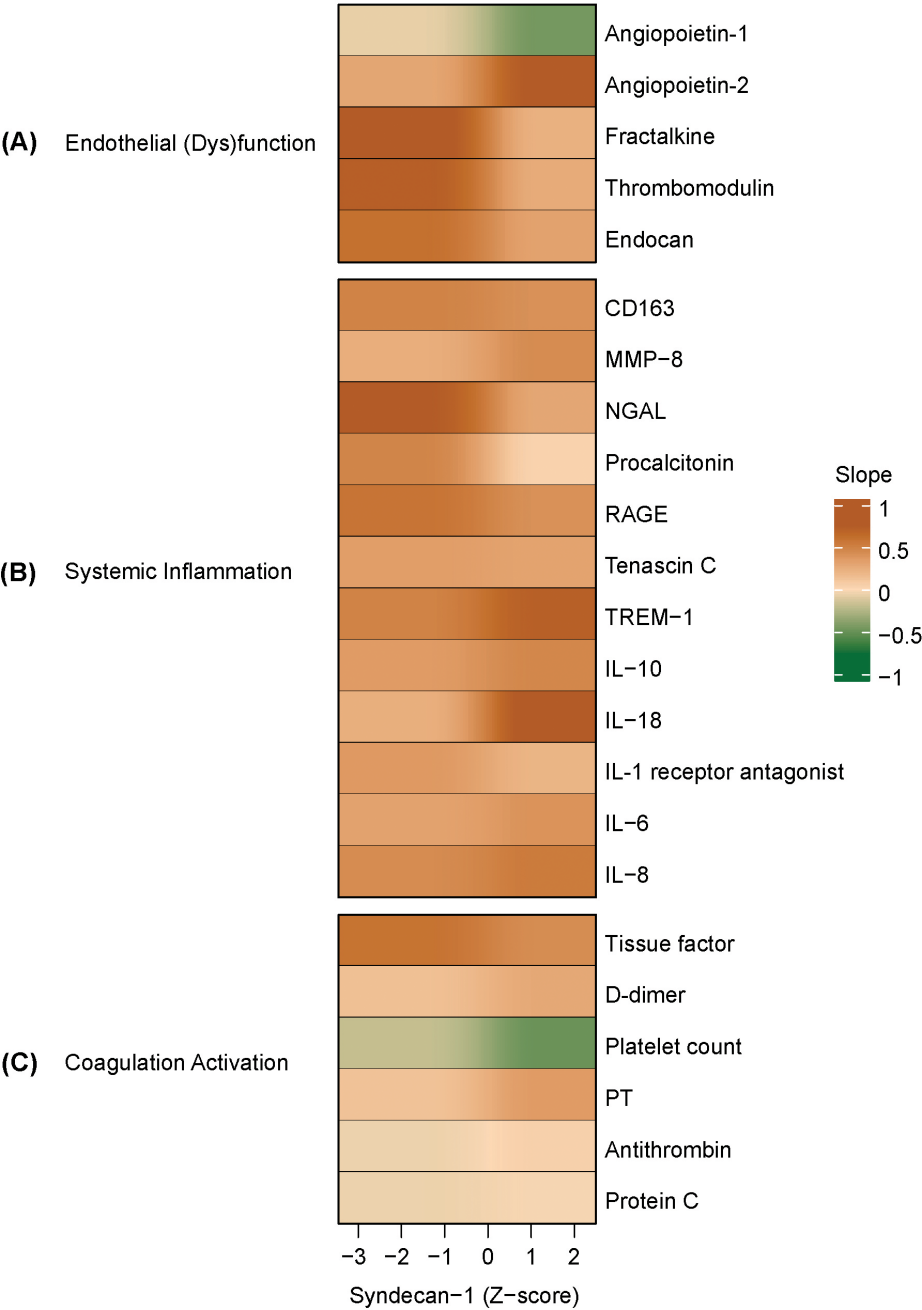
Dynamic slope patterns of host response biomarkers across increasing syndecan-1 levels. Syndecan-1 levels were modelled in relation to three categories of host response biomarkers: **(A)** Endothelial cell (dys)function. **(B)** Systemic inflammation. **(C)** Coagulation activation. All variables were log_10_-transformed and standardized using Z-scores to ensure comparability. For each marker, a restricted cubic spline regression was fitted against syndecan-1 (Z-score), and the fitted curve was evaluated at 100 equally spaced intervals across the observed range. Each slope represents the first derivative of the fitted spline curve, approximating the instantaneous rate of change in biomarker levels per unit change in syndecan-1 Z-score. Green indicates negative (edecreasing) changes; orange indicates positive (increasing) changes, and darker shades reflect faster rates of change.

To further explore how host response biomarkers change across increasing plasma levels of syndecan-1, we standardized the concentrations of all biomarkers using z-scores and then used spline regression to model their associations as a function of syndecan-1. We then calculated the slope on the z-score scale of each fitted curve to describe the direction and rate of change at different syndecan-1 levels. Different patterns emerged: some biomarkers changed at low syndecan-1 levels, while others changed most steeply at higher syndecan-1 concentrations, indicating different fitted-curve patterns (**Figure 3**). In the endothelial domain angiopoietin-1 declined steeply at high syndecan-1 levels, while angiopoietin-2 increased sharply in a similar syndecan-1 range. Considering that the angiopoietin-2 to -1 ratio reflects endothelial barrier function (Maneta et al., 2023; Girardis et al., 2024; van der Poll and Parker, 2020; Curtiaud et al., 2025), these data suggest that higher syndecan-1 levels coincide with a more disturbed angiopoietin profile. In contrast, fractalkine and thrombomodulin, reflecting endothelial activation and injury respectively (Curtiaud et al., 2025; Hoogendijk et al., 2015), displayed sharp rises at lower syndecan-1 levels in this model, plateauing thereafter. In the systemic inflammation domain several markers demonstrated steeper increases at high syndecan-1 concentrations, particularly matrix metalloproteinase (MMP)-8, TREM-1, IL-18, IL-6 and IL-8, suggesting that at least some components of the systemic inflammatory response in BSI become stronger at a certain extent of glycocalyx degradation showed their strongest variation in the higher syndecan-1 range. In the inflammation domain NGAL and procalcitonin showed the steepest rise at relatively low syndecan-1 concentrations. In the coagulation domain a decrease in platelet counts and prolongation of prothrombin time increased most clearly at higher syndecan-1 levels, while the slope of change was modest throughout for D-dimer, antithrombin and protein C. These results highlight the heterogeneous nature of host response patterns associated with (the extent of) glycocalyx degradation.

### Association of syndecan-1 with blood transcriptomes

**Figure 4:**
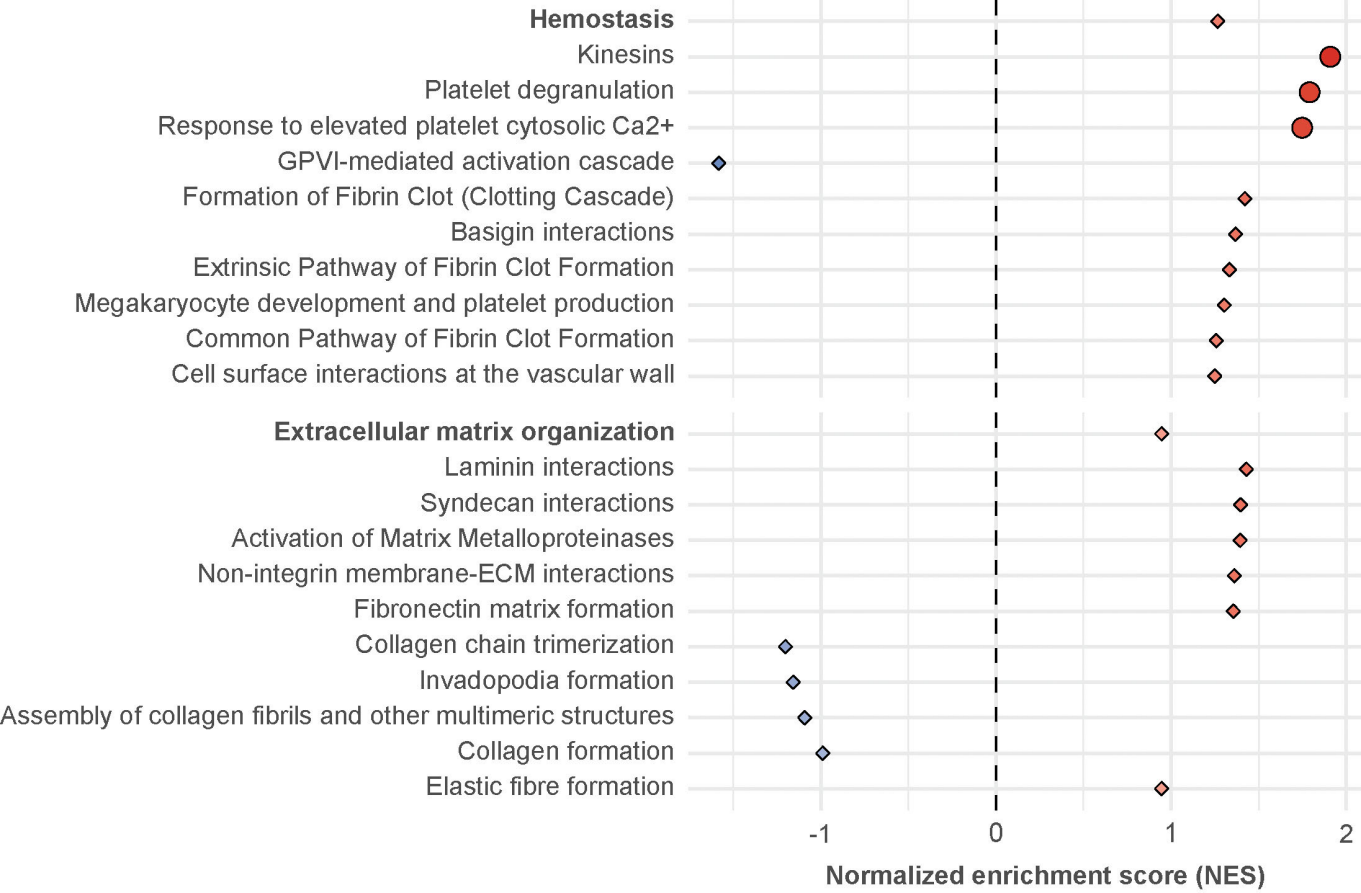
Association of the blood transcriptome with plasma syndecan-1 concentrations. The dot plot displays results from gene set enrichment analysis (GSEA) using Reactome pathways based on genes ranked by correlation with syndecan-1. Pathways are grouped by hierarchy, with bold text indicating parent pathways and regular text indicating child pathways. The x-axis shows the normalized enrichment score (NES). The dot shape indicates statistical significance (circles: False Discovery Rate < 0.05; diamonds: not significant), dot size reflects enrichment magnitude. Color represents direction, with red indicating enrichment at higher syndecan-1 levels and blue indicating enrichment at lower syndecan-1 levels. The top ten enriched child pathways for each parent pathway are shown.

Blood transcriptome data were available in a subgroup of patients (n = 142; **Supplementary Table 8**). We integrated this whole-blood gene expression data to explore if transcriptomic changes were associated with syndecan-1 levels, using syndecan-1 a continuous variable. Considering the known role of the glycocalyx in preserving vascular integrity, and regulating leukocyte adhesion and hemostasis (Uchimido et al., 2019; Iba and Levy, 2019; Iba et al., 2024), we performed GSEA using the Reactome database (Milacic et al., 2024), focusing on pathways related to hemostasis and extracellular matrix organization (**Figure 4**). Several pathways were significantly enriched in patients with high syndecan-1 levels. Within the “Hemostasis” pathway, three pathways were significantly upregulated, which were related to platelet functions, i.e., “Kinesins” (which among other are involved in the transport of cellular components necessary for the development and function of platelets), “Platelet degranulation” and “Response to elevated platelet cytosolic Ca²^+^” (a key signaling event that triggers platelet aggregation and shape change). Within the “Extracellular matrix organization” pathway several pathways showed evidence of positive association with plasma syndecan-1 levels, but none reached statistical significance.

To confirm the robustness of the findings, we performed a supplementary analysis using a tertile-based grouping of syndecan-1 levels. GSEA comparing blood gene expression between the highest and lowest syndecan-1 tertiles was consistent with GSEA findings using syndecan-1 as a continuous variable (**Supplementary Figure 5**).

Finally, to address genes related to syndecan shedding or glycocalyx degradation, we performed an exploratory targeted analysis of selected whole-blood transcripts, including *ADAM17*, *ADAM10*, *HPSE*, *MMP* family members, *ADAMTS1/4*, *HYAL1/2*, *PLG* and *F2*. Thirteen of identified genes of interest were available in the transcriptomic dataset; their expression showed low correlations with plasma syndecan-1 (**Supplementary Table 9**).

## Discussion

The endothelial glycocalyx is crucial for vascular homeostasis by regulating vascular permeability, preventing coagulation and modulating interactions between blood cells and the vessel wall (Uchimido et al., 2019; Iba and Levy, 2019; Moore et al., 2021; Iba et al., 2024). Previous investigations reported associations between elevated circulating syndecan-1 concentrations, as a marker of glycocalyx degradation, and mortality in patients with sepsis (Fernández-Sarmiento et al., 2023). Yet, these earlier studies did not evaluate factors associated with glycocalyx breakdown or associations with other host response changes implicated in sepsis pathogenesis. We sought to address this knowledge gap in a large cohort of critically ill patients with microbiologically proven bacterial BSI. We confirmed that plasma syndecan-1 has a positive association with mortality, which remained after adjusting for possible confounders including disease severity. Almost 20 percent of the variation in syndecan-1 levels was explained by disease severity, as indicated by the SOFA score, while broad pathogen categories were not significantly associated with syndecan-1 levels. A comprehensive analysis of host response domains indicated that syndecan-1 elevation is linked, in differing degrees, with endothelial dysfunction, systemic inflammation and coagulation abnormalities. These results add to our recent study in community-acquired pneumonia, which indicated that glycocalyx disruption is a relatively late phenomenon in the spectrum of host response aberrations in patients with disease severities spanning from mild to severe (Wang et al., 2026).

Many bacteria produce toxins that disrupt the endothelial barrier through mechanisms, such as directly killing endothelial cells or impairing the cytoskeleton and cell-cell junctions (Park et al., 2001, Park et al., 2004; Lubkin and Torres, 2017; Obino and Duménil, 2019). Notable pathogens in this context include *Escherichia coli* (which produces Shiga toxin), *Pseudomonas aeruginosa* (utilizing both type 2 and type 3 secretion systems), *Streptococcus pneumoniae* (producing pneumolysin), and *Staphylococcus aureus* (secreting α-toxin). Additionally, Gram-negative bacteria can release outer membrane vesicles containing endotoxin and various virulence factors that alter endothelial cell function (Lubkin and Torres, 2017). We hypothesized that if the pathogen contributes to glycocalyx degradation during bacterial infection, this effect would be most evident in patients with positive BCs, since the pathogen’s presence in the bloodstream allows direct interaction with the endothelium. However, broad pathogen categories did not explain syndecan-1 variation to a significant extent in syndecan-1 levels to a significant extent, and comparisons between BSI groups revealed no differences in syndecan-1 concentrations. Likely, in ICU patients a variety of damaging factors - including a severe hyperinflammatory state, oxidative stress, ischemia/reperfusion injury, hyperglycemia, fluid overload, electrolyte imbalances, trauma, and surgical interventions – have a large impact on glycocalyx integrity.

Laboratory investigations have indicated interactions between the glycocalyx and biological functions related to the vascular endothelium, inflammation and coagulation (Uchimido et al., 2019; Iba and Levy, 2019; Moore et al., 2021; Iba et al., 2024). We used several approaches to examine these relations in patients *in vivo*. PCA of biomarkers grouped according to pathophysiological domains documented the strongest associated of syndecan-1 with endothelial (dys)function. Restricted cubic spline regression, used to map how biomarker levels varied across the range of syndecan-1 levels, suggested that impairment of barrier function (indicated by decreasing angiopoietin-1 and increasing angiopoietin-2 (Maneta et al., 2023; Girardis et al., 2024; van der Poll and Parker, 2020; Curtiaud et al., 2025)) was most evident at a relatively high degree of glycocalyx damage, whereas other endothelial markers indicating activation (fractalkine) (Hoogendijk et al., 2015) and injury (thrombomodulin) (Curtiaud et al., 2025) showed steep associations already at low syndecan-1 levels. Inflammation markers demonstrated variable patterns relating to increasing syndecan-1 concentrations, with some markers rising at low syndecan-1 (particularly NGAL and to a lesser extent procalcitonin) and many markers with the steepest increase at higher syndecan-1 (particularly soluble TREM-1, IL-18 and MMP-8). These findings are consistent with the concept of close interrelations between inflammation and glycocalyx degradation, with elevated inflammatory markers and markers of endothelial injury increasing in parallel across the syndecan-1 range (Uchimido et al., 2019; Iba and Levy, 2019; Moore et al., 2021). The glycocalyx provides an anticoagulant layer on the endothelium, and its degradation promotes coagulation by impairing this antithrombotic function (Uchimido et al., 2019; Moore et al., 2021). Changes in coagulation markers showed different patterns across the syndecan-1 range. Soluble tissue factor displayed a steep surge at relatively low syndecan-1 levels, whereas D-dimer showed a gradual rise with increasing syndecan-1 levels. Signs of consumptive coagulopathy - a decrease in platelet counts and prolongation of the prothrombin time - became particularly evident at higher syndecan-1 levels, supporting the notion that glycocalyx degradation may further enhance activation of the coagulation system.

Analysis of the blood transcriptome uncovered increased expression of genes related to hemostasis, specifically pointing at enhanced platelet functions in patients with high syndecan-1 levels. Although we did not directly assess platelet functions, these data are in agreement with the capacity of an intact endothelial glycocalyx to inhibit platelet adhesion and activation by masking adhesion molecules and interacting with clotting factors (Uchimido et al., 2019; Iba and Levy, 2019; Moore et al., 2021).

Our findings may have clinical implications. Circulating syndecan-1 could help identify patients with BSI who have more severe glycocalyx injury and associated endothelial, inflammatory and coagulation changes. While plasma syndecan-1 levels may be relevant for risk stratification, we did not assess whether syndecan-1 improves outcome prediction beyond established clinical severity scores. The endothelial glycocalyx has also been proposed as a diagnostic and therapeutic target in sepsis (Uchimido et al., 2019; Iba et al., 2024). However, our data do not establish syndecan-1 as a treatment-guiding biomarker. Prospective studies are needed to determine whether glycocalyx preservation or restoration can modify host responses and/or improve clinical outcomes.

Our study has strengths and limitations. We measured 24 biomarkers and analyzed the blood transcriptome to provide insight into host response pathways implicated in sepsis pathogenesis in a large cohort of critically ill patients with documented BSIs. Yet, our biomarker panel did not include mediators of syndecan-1 shedding. Because plasma was obtained from left-over routine clinical samples, detailed pre-analytical information, including residual platelet content, was not available. We used restricted cubic spline regression to map how biomarker levels varied across the range of syndecan-1 levels; yet, the cross-sectional and observational nature of our analyses precludes establishing temporal and causal relationships between glycocalyx disruption and other host responses. Longitudinal studies with repeated measurements of syndecan-1 and other biomarkers would offer a more dynamic insight into glycocalyx degradation in the progression of severe infection. Treatment-related factors before biomarker sampling were not accounted for, including antibiotic timing and adequacy, source control, fluid resuscitation, vasopressor exposure and other ICU interventions. Although biomarkers were measured early after ICU admission, some treatments may already have been started. Residual confounding by treatment-related factors therefore cannot be excluded. A power calculation was not performed which may have limited the ability to detect smaller effect sizes. Although syndecan-1 is a well-established marker of glycocalyx degradation, it may not fully reflect all dimensions of glycocalyx integrity; future studies combining multiple glycocalyx-associated markers and/or direct visualization methods could afford a more comprehensive evaluation. Moreover, measurement of plasma syndecan-1, while likely derived from the endothelium, does not identify its exact tissue or cellular source. Plasma syndecan-1 levels can be influenced by factors beyond glycocalyx degradation, such as renal function. However, a sensitivity analysis including plasma creatinine and non-renal SOFA did not materially change the interpretation. In addition, our biomarker panel was designed to characterize host-response domains, not to directly assess syndecan-1 sheddases or regulators.

## Conclusion

Protecting the glycocalyx has been proposed as a therapeutic strategy in sepsis, given its central role in maintaining homeostasis across multiple biological pathways. In this study of critically ill patients with BSIs, the causative pathogen had no significant impact on the extent of glycocalyx degradation, which was instead partly determined by disease severity. While most knowledge of glycocalyx function is derived from experimental models, this investigation combined analytical and computational methods in patients *in vivo*, revealing that glycocalyx breakdown is closely associated with systemic disturbances in endothelial function, inflammation, and coagulation.

## Data Availability

The datasets generated and analyzed during the current study are not publicly available because they contain clinical patient-level data, but are available from the corresponding author upon reasonable request and subject to applicable ethical and data-sharing regulations. The **Supplementary Material** contains supporting tables and figures, but not the original patient-level dataset.
