# Supplementary material for: Endothelial glycocalyx injury in bacterial bloodstream infection: biological determinants and association with host response aberrations
Source: Front Cell Infect Microbiol. 2026 Jun 2;16:1821582. doi: 10.3389/fcimb.2026.1821582 (PMC13269403; doi:10.3389/fcimb.2026.1821582)
Supplement: Supplementary file 1 [file SupplementaryFile1.docx]

Supplementary material

**Endothelial glycocalyx injury in bacterial bloodstream infection: biological determinants and association with host response aberrations**

**Hui Wang*^1^**, **Joe M. Butler^1^**, **Erik H. A. Michels^1^**, **Tom D. Y. Reijnders^1^**, **Tjitske S.R. van Engelen^1^**, **Alex F. de Vos^1^**, **Olaf L. Cremer^2^**, **Hessel Peters-Sengers^1,3^**, **Tom van der Poll^1,4^**

*** Corresponding Author:**

hui.nerissa.wang@amsterdamumc.nl

**Table of Contents**

***Supplementary tables***

Table S1 Stratification of biomarkers in host response domains…………………………………………..6

Table S2 Distribution of pathogens………………………………………………………………………………………7

Table S3 Baseline characteristics and admission diagnoses of non-infectious control patients……………………………………………………………………………………………...………………..………………9

Table S4 Association between plasma syndecan-1 and 30-day mortality……………………11

Table S5 Baseline characteristics and clinical outcomes of critically ill patients with bloodstream infection stratified by plasma syndecan-1 tertiles upon intensive care unit admission……………………………………………………………………………………………...……………………………12

Table S6 Contribution of biomarkers to principal components 1 and 2 of each host response domain……………………………………………………………………………………………...……………………………….14

Table S7 Plasma biomarker concentrations……………………………………...…………………………………15

Table S8 Baseline characteristics of patients with available blood transcriptome data…………16

Table S9 Targeted analysis of expression of genes in whole blood implicated in syndecan-1 shedding and glycocalyx degradation…………………………………………………………18

***Supplementary figures***

Figure S1 Kaplan-Meier 30-Day Survival Curve Stratified by Syndecan-1 Tertiles…….…….…19

Figure S2 Sensitivity analysis adjusted for plasma creatinine……………………………………………….20

Figure S3 PCA of host response pathways across Syndecan-1 tertiles………………………………….21

Figure S4 Sensitivity analysis of the coagulation domain PCA using non-imputed antithrombin and protein C measurements……………………………………...………………………………………………………..22

Figure S5 Differentially expressed genes and pathway enrichment results comparing the lowest to the highest syndecan-1 tertile……………………………………...…………………………………………………..23

**Supplementary materials**

**Definitions**

Malignancy was defined as a medical history of either non-metastatic solid tumor, metastatic malignancy or hematologic malignancy. Renal insufficiency was defined as a history of chronic renal insufficiency or chronic intermitted hemodialysis or continuous ambulatory peritoneal dialysis. Immunosuppression was described as a history of organ transplantation, primary or secondary immunodeficiency, or long-term use of immunosuppressive agents. Shock was defined by the use of noradrenaline for hypotension in a dose of more than 0.1 μg/kg/min during at least 50% of the ICU day (van Vught et al., 2016). Acute respiratory distress syndrome and acute kidney injury were defined using strict preset criteria (Bellomo et al., 2004, Bernard et al., 1994).

**Luminex biomarker assay**

A custom discovery Luminex assay was designed using R&D Systems reagents to simultaneously quantify 20 biomarkers in EDTA plasma ([Human Luminex® Discovery Assay LXSAHM: R&D Systems](https://www.rndsystems.com/products/human-luminex-discovery-assay_lxsahm)). For syndecan-1 detection, the assay utilized magnetic color-coded microspheres conjugated with a monoclonal capture antibody paired with a biotinylated monoclonal detection antibody and streptavidin-phycoerythrin for fluorescent detection on Luminex platforms, using a recombinant human syndecan-1 standard (sensitivity 15.6 pg/mL; intra- and inter-assay coefficient of variation variability <10%). Antithrombin and protein C were also measured by Luminex technology, yet in a separate assay done on samples collected during the first 2,5 years of the MARS project. All samples were processed according to R&D Systems protocols, with data acquired using a Luminex analyzer and analyzed via standard curve interpolation.

**Transcriptomic Profiling and Analysis**

Total RNA was extracted with the QIAcube system and Blood mRNA kit (Qiagen, Venlo, the Netherlands). RNA quality was checked on the Agilent Bioanalyzer (Agilent, Santa Clara, CA, USA); all samples had RNA integrity numbers (RIN) above six (Schroeder et al., 2006). RNA concentration was measured with the Qubit® 2.0 Fluorometer (Life Technologies, Carlsbad, CA, USA). Libraries were prepared using the KAPA RNA HyperPrep Kit with RiboErase (Roche). For the RNA sequencing (RNA-seq) platform, sequencing was done on the Illumina HiSeq4000 (Illumina, San Diego, CA, USA), producing 50 bp single-end reads. Read quality was assessed with FastQC (v0.11.5). Trimmomatic (v0.39) was used to remove Illumina adapters, low-quality bases, and reads with ambiguous nucleotides. After trimming, high-quality reads were aligned to the GRCh38 reference genome (Ensembl release 84) with HISAT2 (v2.2.0). Gene counts were obtained with HTSeq (Anders et al., 2015). Gene expression from U219 microarrays (Affymetrix) and GeneChip Human Transcriptome Array (HTA) 2.0 (Thermo Fisher) was processed using robust multiarray averaging (RMA) normalization and background correction, as previously described (Scicluna et al., 2015, Scicluna et al., 2020).

**Statistical Analysis**

To assess the nonlinear association between syndecan-1 levels and 30-day mortality, we used restricted cubic spline logistic regression with three default quantile-based knots (Ahmed, 2016). Overall and nonlinearity significance was tested using Wald-type analysis of variance (ANOVA). Odds ratios and 95% confidence intervals were derived using the Predict function from the *rms* package, with variables kept on their natural scale; for spline plots, predictions were centered at the sample median so that the odds ratio equals 1 at this reference point (Harrell Jr et al., 2017). Differences in syndecan-1 levels across pathogen groups were tested with the Kruskal-Wallis test. To identify determinants of syndecan-1 levels, we built multivariable linear regression models with log₁₀-transformed syndecan-1 as the outcome (Grömping, 2007). Predictors included pathogen type, infection site, SOFA score, demographics (age, sex), and comorbidities. We performed ANOVA to decompose variance and visualized each variable’s relative contribution using bar plots. We treated the relationships between syndecan-1 and many biomarkers as potentially nonlinear. To explore host response changes across the syndecan-1 gradient, all biomarkers were standardized using Z-score. We modelled each using lm(marker ~ rcs(syndecan-1, 3)), then computed the first derivatives (slopes) across 100 equally spaced Syndecan-1 values. Slopes were winsorized at the 95th percentile to limit outlier effects. Heatmaps were generated with *ComplexHeatmap*, with markers grouped by functional domains (endothelial cell (dys)function, systemic inflammation, coagulation activation). We also fitted log-log models (log₁₀-transformed syndecan-1 and biomarkers), back-transformed the predictions, and visualized the relationships on the original scale. All spline fits used log₁₀-transformed values. Predictions and confidence intervals were back-transformed using (10^x − 1) to return to the original scale. Biomarker concentrations were winsorized at the 99th percentile to reduce the influence of extreme values. Analyses and plots were conducted in R using the *rms*, *ggplot2*, *patchwork*, *dplyr*, *scales*, and *tidy* packages.

**Missingness**

Missingness was highest for antithrombin and protein C (both 19.7%) and fractalkine (8.0%). Other variables, including matrix metalloproteinase-8, platelet counts, prothrombin time, heart rate, respiratory rate, core temperature, and systolic pressure, had 1–6% missingness. Missing data were imputed using the mice package with CART (Van Buuren and Groothuis-Oudshoorn, 2011).

**References**

Ahmed, S. 2016. Regression Modeling Strategies with Applications to Linear Models, Logistic and

Ordinal Regression, and Survival Analysis. Springer.

Anders, S., Pyl, P. T.,Huber, W. (2015). HTSeq—a Python framework to work with high-throughput

sequencing data. *Bioinformatics*. 31, 166–169. doi: 10.1093/bioinformatics/btu638

Bellomo, R., Ronco, C., Kellum, J. A., Mehta, R. L., Palevsky, P.,workgroup, A. (2004). Acute renal

failure–definition, outcome measures, animal models, fluid therapy and information technology needs:

the Second International Consensus Conference of the Acute Dialysis Quality Initiative (ADQI) Group.

*Crit. Care.* 8, R204. doi: 10.1186/cc2872

Bernard, G. R., Artigas, A., Brigham, K. L., Carlet, J., Falke, K., Hudson, L., et al. (1994). The

American-European Consensus Conference on ARDS. Definitions, mechanisms, relevant outcomes, and

clinical trial coordination. *Am J Respir Crit Care Med.* 149, 818–824. doi: 10.1164/ajrccm.149.3.7509706

Grömping, U. (2007). Relative importance for linear regression in R: the package relaimpo. *J. Stat. Softw.* 17,

127. doi: 10.18637/jss.v017.i01

Harrell Jr, F. E., Harrell Jr, M. F. E.,Hmisc, D. (2017). rms: Regression Modeling Strategies. R package version

5.1-1.

Schroeder, A., Mueller, O., Stocker, S., Salowsky, R., Leiber, M., Gassmann, M., et al. (2006). The RIN:

an RNA integrity number for assigning integrity values to RNA measurements. *BMC Mol Biol.* 7, 3. doi:

10.1186/1471-2199-7-3

Scicluna, B. P., Klein Klouwenberg, P. M., van Vught, L. A., Wiewel, M. A., Ong, D. S., Zwinderman, A.

H., et al. (2015). A molecular biomarker to diagnose community-acquired pneumonia on intensive

care unit admission. *Am J Respir Crit Care Med*. 192, 826–835. doi: 10.1164/rccm.201502-0355OC

Scicluna, B. P., Uhel, F., van Vught, L. A., Wiewel, M. A., Hoogendijk, A. J., Baessman, I., et al. (2020).

The leukocyte non-coding RNA landscape in critically ill patients with sepsis. *eLife*. 9, e58597. doi:

10.7554/eLife.58597

Van Buuren, S.,Groothuis-Oudshoorn, K. (2011). mice: Multivariate imputation by chained equations

in R. *J. Stat. Softw.* 45, 1–67. doi: 10.18637/jss.v045.i03

van Vught, L. A., Klouwenberg, P. M. K., Spitoni, C., Scicluna, B. P., Wiewel, M. A., Horn, J., et al.

(2016). Incidence, risk factors, and attributable mortality of secondary infections in the intensive

care unit after admission for sepsis. *JAMA*. 315, 1469–1479. doi: 10.1001/jama.2016.2691

**Tables**

Table S1: Stratification of biomarkers in host response domains

| Endothelial cell activation and dysfunction | Systemic inflammation and organ damage | Coagulation activation |
| --- | --- | --- |
| Angiopoietin-1 | CD163 | Tissue Factor |
| Angiopoietin-2 | IL-10 | D-dimer |
| Fractalkine | IL-18 | Platelet count |
| Thrombomodulin | IL-1 receptor antagonist | PT |
| Endocan | IL-6 | Antithrombin |
| Syndecan-1 | IL-8 | Protein C |
|  | MMP-8 |  |
|  | NGAL |  |
|  | Procalcitonin |  |
|  | RAGE |  |
|  | Tenascin C |  |
|  | TREM1 |  |
| Abbreviations: CD: cluster of differentiation 163; IL: Interleukin; MMP: matrix metalloproteinase; NGAL: neutrophil gelatinase-associated lipocalin; PT: prothrombin time; RAGE: receptor for advanced glycation end-products; TREM: triggering receptor expressed on myeloid cells. | | |

Table S2: Distribution of pathogens

**Monomicrobial bloodstream infections (N = 165)**

| **Species** | N |
| --- | --- |
| ***Escherichia coli*** | **38** |
| ***Streptococcus species*** | **34** |
| *Streptococcus pneumoniae* | 13 |
| Group A | 6 |
| Group B | 1 |
| Group G | 1 |
| *Streptococcus viridans group* | 6 |
| *Streptococcus* species (unspecified) | 7 |
| ***Staphylococcus aureus*** | **23** |
| ***Klebsiella species*** | **14** |
| *Klebsiella pneumoniae* | 13 |
| *Klebsiella oxytoca* | 1 |
| ***Pseudomonas aeruginosa*** | **8** |
| ***Enterococcus species*** | **25** |
| *Enterococcus faecium* | 13 |
| *Enterococcus faecalis* | 9 |
| *Enterococcus species* (unspecified) | 3 |
| ***Enterobacter species*** | **8** |
| *Enterobacter cloacae* | 6 |
| *Enterobacter aerogenes* | 1 |
| *Enterobacter species* (unspecified) | 1 |
| ***Other*** | **15** |
| *Clostridium species* | 3 |
| *Bacteroides fragilis* | 2 |
| *Citrobacter koseri* | 2 |
| *Clostridium bifermentans* | 1 |
| *Clostridium perfringens* | 1 |
| *Bacteroides thetaiotaomicron* | 1 |
| *Proteus mirabilis* | 1 |
| *Serratia marcescens* | 1 |
| *Salmonella group B* | 1 |
| *Haemophilus influenzae* | 1 |
| *Neisseria meningitidis* | 1 |

**Mixed bloodstream infections (N = 23)**

| Species | N |
| --- | --- |
| ***Mixed Gram-negative*** | **5** |
| *Citrobacter koseri, Klebsiella oxytoca* | 1 |
| *Escherichia coli, Klebsiella pneumoniae* | 1 |
| *Enterobacter cloacae, Klebsiella pneumoniae* | 1 |
| *Escherichia coli, Proteus mirabilis* | 1 |
| *Escherichia coli, Pseudomonas aeruginosa* | 1 |
| ***Mixed Gram-positive*** | **2** |
| *Staphylococcus aureus, Streptococcus* group B *(Str. agalactiae)* | 1 |
| *Clostridium* species*, Staphylococcus aureus* | 1 |
| ***Mixed Other*** | **16** |
| *Enterobacter asburiae, Enterococcus faecium* | 1 |
| *Enterococcus faecium, Enterococcus faecalis, Pseudomonas aeruginosa* | 1 |
| *Escherichia coli, Eggerthella lenta* | 1 |
| *Enterococcus* species*, Klebsiella pneumoniae* | 1 |
| *Enterococcus faecium, Bacteroides thetaiotaomicron* | 1 |
| *Escherichia coli, Enterococcus faecium* | 1 |
| *Enteroccus faecium, Enterococcus* species, *Escherichia coli* | 1 |
| *Streptococcus,* unspecified gram-negative bacterium | 1 |
| *Abiotrophia* species*, Fusobacterium necrophorum* | 1 |
| *Enterococcus faecium, Escherichia coli, Klebsiella oxytoca* | 1 |
| *Aeromonas* species*, Enterococcus faecalis, Escherichia coli* | 1 |
| *Morganella morganii subsp. morganii, Proteus mirabilis, Streptococcus haemolyticus* group G | 1 |
| *Enterococcus* species*, Escherichia coli, Klebsiella oxytoca* | 1 |
| *Enterobacter aerogenes, Enterococcus avium, Escherichia coli* | 1 |
| *Enterococcus faecium, Escherichia coli, Klebsiella pneumoniae, Enterobacter cloacae* | 1 |
| *Enterococcus* species*, Escherichia coli, Klebsiella pneumoniae, Streptococcus viridans* | 1 |

Table S3: Baseline characteristics and admission diagnoses of non-infectious control patients

| n | 53 |
| --- | --- |
| Syndecan-1 (pg/ml), median [IQR] | 5750.7 [3719.0, 9366.4] |
| Demographics |  |
| Age, years, median [IQR] | 64.00 [48.00, 73.00] |
| Sex, male, n (%) | 34 (64.2) |
| Body Mass Index, median [IQR] | 24.47 [21.96, 27.06] |
| Comorbidities |  |
| Chronic obstructive pulmonary disease, n (%) | 6 (11.3) |
| Congestive heart failure, n (%) | 2 (3.8) |
| Prior myocardial infarction, n (%) | 3 (5.7) |
| Cerebrovascular disease, n (%) | 1 (1.9) |
| (Prior) malignancy, n (%) | 3 (5.7) |
| Immunosuppression, n (%) | 5 (9.4) |
| Chronic kidney disease, n (%) | 7 (13.2) |
| Diabetes, n (%) | 11 (20.8) |
| Severity on admission |  |
| APACHE IV, median [IQR] | 71.00 [48.00, 95.00] |
| SOFA score, median [IQR] | 7.00 [3.00, 9.00] |
| Shock, n (%) | 16 (30.2) |
| Acute kidney injury, n (%) | 18 (34.0) |
| Acute respiratory distress syndrome, n (%) | 1 (1.9) |
| Routine laboratory markers |  |
| Platelet counts (x10^9^/L), median [IQR] | 184.00 [126.00, 229.00] |
| Leukocyte counts (x10^9^/L), median [IQR] | 12.50 [9.50, 16.00] |
| Creatinine (µmol/L), median [IQR] | 102.00 [64.00, 170.00] |
| Bilirubin (µmol/L), median [IQR] | 11.00 [6.00, 21.00] |
| Clinical course |  |
| Length of hospital stay, days, median [IQR] | 8.15 [3.54, 15.98] |
| ICU mortality, n (%) | 10 (18.9) |
| 30-day mortality, n (%) | 11 (20.8) |
| 90-day mortality, n (%) | 15 (28.3) |
| Admission diagnoses (%) |  |
| Acid-base electrolyte disturbance | 1 (1.9) |
| Acute neurological disease (cerebrovascular accident, haemorrhage) | 6 (11.3) |
| Cardiac arrest | 6 (11.3) |
| Cardiac failure | 3 (5.7) |
| Complications after kidney transplantation | 1 (1.9) |
| Diabetic ketoacidosis | 2 (3.8) |
| Dissecting aortic aneurysm | 3 (5.7) |
| Drug overdose | 1 (1.9) |
| Endarterctomy | 1 (1.9) |
| Gastrointestinal surgery | 2 (3.8) |
| Hepatic failure | 1 (1.9) |
| Pulmonary embolism | 1 (1.9) |
| Respiratory failure | 11 (20.8) |
| Retroperitoneal lymph node dissection | 1 (1.9) |
| Trauma | 2 (3.8) |
| Unspecified | 11 (20.8) |
| Abbreviations: APACHE: acute physiology and chronic health evaluation. SOFA: sequential organ failure assessment. Continuous data are displayed as median [interquartile range] and compared using Kruskal–Wallis test; categorical data are displayed as count (percentage) and compared using Fisher’s exact test. | |

Table S4: Association between plasma syndecan-1 and 30-day mortality

| Model | Contrast | Syndecan-1 level, pg/mL | OR (95% CI) | p value |
| --- | --- | --- | --- | --- |
| Unadjusted | 75th percentile vs median | 19170.7 vs 13128.7 | 1.54 (1.25-1.91) | <0.001 |
| Unadjusted | 90th percentile vs median | 27923.5 vs 13128.7 | 2.73 (1.65-4.52) | <0.001 |
| Adjusted | 75th percentile vs median | 19170.7 vs 13128.7 | 1.29 (1.00-1.65) | 0.476 |
| Adjusted | 90th percentile vs median | 27923.5 vs 13128.7 | 2.19 (1.20-4.00) | 0.011 |

Table S5: Baseline characteristics and clinical outcomes of critically ill patients with bloodstream infection stratified by plasma syndecan-1 tertiles upon intensive care unit admission

|  | Lowest tertile | Middle tertile | Highest tertile | p-value |  |
| --- | --- | --- | --- | --- | --- |
| n | 63 | 62 | 63 |  |  |
| Syndecan-1 (pg/ml), median [IQR] | 5528.08 [3984.65, 7645.69] | 13128.65 [11216.63, 14917.53] | 22197.07 [19066.98, 29482.88] | <0.001 |  |
| Demographics |  |  |  |  |  |
| Age, years, median [IQR] | 62 [53, 69] | 65 [54, 72] | 62 [53, 72] | 0.705 |  |
| Sex, male, n (%) | 40 (63.5) | 37 (59.7) | 42 (66.7) | 0.719 |  |
| Body Mass Index, median [IQR] | 23.95 [22.36, 26.97] | 24.69 [23.41, 26.60] | 24.44 [21.23, 27.45] | 0.674 |  |
| Comorbidities |  |  |  |  |  |
| Chronic obstructive pulmonary disease,, n (%) | 8 (12.7) | 10 (16.1) | 3 (4.8) | 0.117 |  |
| Congestive heart failure, n (%) | 3 (4.8) | 4 (6.5) | 0 (0.0) | 0.141 |  |
| Prior myocardial infarction, n (%) | 6 (9.5) | 7 (11.3) | 4 (6.3) | 0.621 |  |
| Cerebrovascular disease, n (%) | 6 (9.5) | 6 (9.7) | 4 (6.3) | 0.752 |  |
| (Prior) malignancy, n (%) | 15 (23.8) | 19 (30.6) | 19 (30.2) | 0.637 |  |
| Immunosuppression, n (%) | 10 (15.9) | 10 (16.1) | 18 (28.6) | 0.128 |  |
| Chronic kidney disease, n (%) | 10 (15.9) | 12 (19.4) | 12 (19.0) | 0.854 |  |
| Diabetes, n (%) | 10 (15.9) | 15 (24.2) | 12 (19.0) | 0.498 |  |
| Severity on admission |  |  |  |  |  |
| APACHE IV, median [IQR] (median [IQR]) | 65.00 [57.00, 88.50] | 85.50 [73.00, 105.75] | 101.00 [75.50, 123.50] | <0.001 |  |
| SOFA score, median (median [IQR]) | 7.00 [5.00, 8.00] | 9.00 [7.00, 11.00] | 10.00 [8.00, 14.00] | <0.001 |  |
| Shock, n (%) | 17 (27.0) | 30 (48.4) | 28 (44.4) | 0.034 |  |
| Acute kidney injury, n (%) | 22 (34.9) | 32 (51.6) | 41 (65.1) | 0.003 |  |
| Acute respiratory distress syndrome, n (%) | 3 (4.8) | 2 (3.2) | 4 (6.3) | 0.716 |  |
| Routine laboratory markers |  |  |  |  |  |
| Platelet counts (x10^9^/L) ,median [IQR] | 162 [115, 254] | 140 [64, 220] | 93 [41, 210] | 0.005 |  |
| Leukocyte counts (x10^9^/L) ,median [IQR] | 16.6 [11.7, 23.2] | 11.9 [7.7, 21.6] | 12.6 [5.2, 20.3] | 0.075 |  |
| Creatinine (µmol/L) ,median [IQR] | 124 [80, 162] | 156 [106, 256] | 159 [122, 247] | 0.002 |  |
| Bilirubin (µmol/L) ,median [IQR] | 12 [8, 19] | 18 [9, 35] | 28 [12, 55] | 0.004 |  |
| Pathogen group* |  |  |  | 0.221 |  |
| *Escherichia coli,* n (%) | 12 (19.0) | 10 (16.1) | 16 (25.4) |  |  |
| *Enterobacter,* n (%) | 1 (1.6) | 4 (6.5) | 3 (4.8) |  |  |
| *Enterococcus species,* n (%) | 6 (9.5) | 10 (16.1) | 9 (14.3) |  |  |
| *Klebsiella species,* n (%) | 4 (6.3) | 6 (9.7) | 4 (6.3) |  |  |
| *Pseudomonas aeruginosa,* n (%) | 4 (6.3) | 1 (1.6) | 3 (4.8) |  |  |
| *Staphylococcus* *aureus,* n (%) | 6 (9.5) | 7 (11.3) | 10 (15.9) |  |  |
| *Streptococcus* species*,* n (%) | 19 (30.2) | 7 (11.3) | 8 (12.7) |  |  |
| Other*,* n (%) | 6 (9.5) | 7 (11.3) | 2 (3.2) |  |  |
| Mixed*,* n (%) | 5 (7.9) | 10 (16.1) | 8 (12.7) |  |  |
| Source of infection^#^ |  |  |  | 0.749 |  |
| Abdominal, n (%) | 13 (20.6) | 16 (25.8) | 22 (34.9) |  |  |
| Cardiovascular, n (%) | 5 (7.9) | 4 (6.5) | 7 (11.1) |  |  |
| Central nervous system, n (%) | 4 (6.3) | 2 (3.2) | 1 (1.6) |  |  |
| Respiratory, n (%) | 13 (20.6) | 9 (14.5) | 10 (15.9) |  |  |
| Skin, n (%) | 10 (15.9) | 9 (14.5) | 7 (11.1) |  |  |
| Urinary, n (%) | 11 (17.5) | 12 (19.4) | 7 (11.1) |  |  |
| Other, n (%) | 6 (9.5) | 6 (9.7) | 5 (7.9) |  |  |
| Unknown, n (%) | 1 (1.6) | 4 (6.5) | 4 (6.3) |  |  |
| Clinical course |  |  |  |  |  |
| Length of hospital stay, days, median [IQR] | 15.6 [8.3, 30.9] | 16.8 [9.5, 43.4] | 16.3 [3.6, 43.2] | 0.542 |  |
| ICU mortality, n (%) | 6 (9.5) | 15 (24.2) | 26 (41.3) | <0.001 |  |
| 30-day mortality, n (%) | 10 (15.9) | 21 (33.9) | 30 (47.6) | 0.001 |  |
| 90-day mortality, n (%) | 16 (25.4) | 31 (50.0) | 33 (52.4) | 0.003 |  |
| * For detailed overview of pathogens see Table S2. ^#^Patients can have multiple sources of infection, and percentages represent the number of each source out of the BSI group sizes. Abbreviations: APACHE: acute physiology and chronic health evaluation; SOFA: sequential organ failure assessment. Continuous data are displayed as median [interquartile range] and compared using Kruskal–Wallis test; categorical data are displayed as count (percentage) and compared using Fisher’s exact test. | | | | |  |

Table S6: Contribution of biomarkers to principal components 1 and 2 of each host response domain

| Endothelial cell activation and dysfunction | | | Systemic inflammation and organ damage | | | Coagulation activation | | |
| --- | --- | --- | --- | --- | --- | --- | --- | --- |
|  | PC1 | PC2 |  | PC1 | PC2 |  | PC1 | PC2 |
| Angiopoietin-1 | 16.66 | 26.92 | CD163 | 6.74 | 5.32 | Tissue factor | 5.76 | 34.5 |
| Angiopoietin-2 | 22.34 | 7.81 | IL-10 | 10.13 | 12.41 | D-dimer | 0.01 | 10.31 |
| Fractalkine | 22.24 | 20.07 | IL-18 | 6.73 | 7.09 | Platelet count | 21.24 | 9.77 |
| Thrombomodulin | 21.21 | 7.38 | IL-1 receptor antagonist | 8.91 | 12.77 | PT | 29.94 | 5.05 |
| Endocan | 17.56 | 37.82 | IL-6 | 10.72 | 17.02 | Antithrombin | 15.79 | 24.69 |
|  |  |  | IL-8 | 10.81 | 8.6 | Protein C | 27.24 | 15.69 |
|  |  |  | MMP-8 | 5.53 | 0.11 |  |  |  |
|  |  |  | NGAL | 11.48 | 1.15 |  |  |  |
|  |  |  | Procalcitonin | 5.84 | 0.83 |  |  |  |
|  |  |  | RAGE | 7.73 | 9.87 |  |  |  |
|  |  |  | Tenascin C | 4.49 | 19.9 |  |  |  |
|  |  |  | TREM-1 | 10.87 | 4.93 |  |  |  |
| The top three contributing biomarkers for each principal component score are highlighted in bold. Abbreviations: see legend of Table S1. | | | | | | | | |

Table S7: Plasma biomarker concentrations

All biomarker concentrations are expressed as pg/ml and reported as median [IQR]. For abbreviations see Table S1.

|  | Lowest tertile | Middle tertile | Highest tertile | p |
| --- | --- | --- | --- | --- |
| n | 63 | 62 | 63 |  |
| Syndecan-1 | 5528.1 [3984.7, 7645.7] | 13128.7 [11216.6, 14917.5] | 22197.1 [19067.0, 29482.9] | <0.001 |
| **Endothelial (dys)function** |  |  |  |  |
|  |  |  |  |  |
| Angiopoietin-1 | 2239.3 [1365.6, 3423.6] | 2311.2 [1086.6, 3418.7] | 1419.4 [750.6, 3053.5] | 0.013 |
| Angiopoietin-2 | 6498.8 [3385.5, 11055.0] | 10208.5 [5877.6, 13940.6] | 13479.7 [7809.8, 22253.6] | <0.001 |
| Fractalkine | 2479.2 [1404.4, 3646.4] | 3963.1 [2963.3, 4849.5] | 4598.7 [3198.8, 7339.0] | <0.001 |
| Thrombomodulin | 5361.4 [4166.8, 6834.1] | 7905.3 [5119.3, 10270.7] | 8614.1 [6319.8, 11521.5] | <0.001 |
| Endocan | 1024.3 [435.0, 1452.5] | 1738.8 [976.5, 3160.3] | 2519.4 [1276.7, 5054.7] | <0.001 |
| **Systemic inflammation** |  |  |  |  |
| CD163 | 566938.9 [298625.2, 1028398.7] | 857029.4 [508948.5, 1496586.5] | 1275709.9 [840391.5, 2212470.2] | <0.001 |
| IL-10 | 16.4 [5.6, 47.3] | 42.3 [20.6, 120.5] | 47.1 [22.1, 246.1] | <0.001 |
| IL-18 | 307.4 [192.7, 404.9] | 358.7 [245.6, 462.3] | 583.3 [381.3, 1101.9] | <0.001 |
| IL-1 receptor antagonist | 12760.9 [3878.1, 27396.6] | 14420.0 [5004.0, 66967.6] | 19447.0 [10048.6, 66967.6] | 0.078 |
| IL-6 | 193.5 [59.7, 1359.3] | 1063.2 [104.4, 5665.5] | 1248.5 [231.1, 6504.4] | 0.002 |
| IL-8 | 25.0 [13.9, 108.9] | 116.5 [36.7, 806.7] | 316.8 [63.3, 2901.6] | <0.001 |
| MMP-8 | 17449.8 [6631.6, 31337.4] | 23869.5 [6713.6, 104250.1] | 60150.1 [26155.3, 102356.6] | <0.001 |
| NGAL | 200026.3 [103175.1, 302784.8] | 300399.5 [197953.5, 510799.9] | 378826.8 [255688.3, 560282.5] | <0.001 |
| Procalcitonin | 6811.5 [1741.1, 17378.6] | 8371.5 [3325.5, 29088.5] | 7860.5 [6448.3, 17684.8] | 0.137 |
| RAGE | 2001.0 [1537.3, 3108.0] | 4352.2 [2453.5, 7085.5] | 4215.4 [2519.0, 8502.6] | <0.001 |
| Tenascin C | 15206.2 [12337.3, 18928.1] | 18083.8 [14412.2, 24982.7] | 21902.4 [15586.5, 33058.4] | <0.001 |
| TREM1 | 277.6 [190.5, 417.8] | 531.7 [362.6, 831.7] | 650.2 [414.4, 1150.1] | <0.001 |
| **Coagulation activation** |  |  |  |  |
| Tissue Factor | 29.1 [23.7, 40.7] | 44.1 [31.1, 59.0] | 56.6 [39.7, 77.8] | <0.001 |
| D-dimer | 2836263.4 [2455458.3, 3244565.1] | 2760914.3 [2078975.5, 3601844.9] | 3217102.0 [2475654.1, 4420234.8] | 0.041 |
| Platelet count | 162.0 [114.5, 254.0] | 139.5 [63.5, 219.5] | 93.0 [41.0, 210.0] | 0.005 |
| PT | 17.0 [13.9, 20.9] | 16.1 [13.4, 20.8] | 19.1 [15.6, 25.5] | 0.007 |
| Antitrombin | 603317.6 [363303.8, 793102.8] | 600955.2 [393798.5, 845980.0] | 607819.2 [487398.6, 811409.6] | 0.881 |
| Protein C | 106654.9 [87478.8, 146153.3] | 107237.2 [96716.0, 133688.8] | 106228.8 [93431.9, 123391.1] | 0.693 |

Table S8: Baseline characteristics of patients with available blood transcriptome data

| n | 142 |
| --- | --- |
| Syndecan-1 (pg/ml), median [IQR] | 12799.11 [6806.30, 18513.90] |
| Demographics |  |
| Age, years, median [IQR] | 63.00 [54.00, 71.00] |
| Sex, male, n (%) | 88 (62.0) |
| Body Mass Index, median [IQR] | 24.49 [22.41, 27.15] |
| Comorbidities |  |
| Chronic obstructive pulmonary disease, n (%) | 18 (12.7) |
| Congestive heart failure, n (%) | 7 (4.9) |
| Prior myocardial infarction, n (%) | 11 (7.7) |
| Cerebrovascular disease, n (%) | 14 (9.9) |
| (Prior) malignancy, n (%) | 36 (25.4) |
| Immunosuppression, n (%) | 26 (18.3) |
| Chronic kidney disease, n (%) | 26 (18.3) |
| Diabetes, n (%) | 31 (21.8) |
| Severity on admission |  |
| APACHE IV, median [IQR] | 84.00 [63.00, 102.00] |
| SOFA score, median [IQR] | 8.00 [6.00, 10.00] |
| Shock, n (%) | 53 (37.3) |
| Acute kidney injury, n (%) | 72 (50.7) |
| Acute respiratory distress syndrome, n (%) | 6 (4.2) |
| Routine laboratory markers |  |
| Platelet counts (x10^9^/L), median [IQR] | 146.00 [83.50, 235.50] |
| Leukocyte counts (x10^9^/L), median [IQR] | 14.40 [8.30, 22.40] |
| Creatinine (µmol/L), median [IQR] | 141.00 [106.00, 199.00] |
| Bilirubin (µmol/L), median [IQR] | 18.00 [9.00, 35.50] |
| Pathogen group* |  |
| *Escherichia coli,* n (%) | 26 (18.3) |
| *Enterobacter ,* n (%) | 5 (3.5) |
| *Enterococcus species,* n (%) | 18 (12.7) |
| *Klebsiella* *species,* n (%) | 10 (7.0) |
| *Pseudomonas aeruginosa,* n (%) | 6 (4.2) |
| *Staphylococcus aureus,* n (%) | 18 (12.7) |
| *Streptococcus* *species,* n (%) | 29 (20.4) |
| Other, n (%) | 12 (8.5) |
| Mixed, n (%) | 18 (12.7) |
| Source of infection^#^ |  |
| Abdominal, n (%) | 41 (28.9) |
| Cardiovascular, n (%) | 13 (9.2) |
| Central nervous system, n (%) | 6 (4.2) |
| Respiratory, n (%) | 22 (15.5) |
| Skin, n (%) | 18 (12.7) |
| Urinary, n (%) | 21 (14.8) |
| Other, n (%) | 16 (11.3) |
| Unknown, n (%) | 5 (3.5) |
| Clinical course |  |
| Length of hospital stay, days, median [IQR] | 16.29 [8.84, 42.42] |
| ICU mortality, n (%) | 32 (22.5) |
| 30-day mortality, n (%) | 42 (29.6) |
| 90-day mortality, n (%) | 54 (38.0) |
| *For detailed overview of pathogens see Table S2. Abbreviations: APACHE: acute physiology and chronic health evaluation. ^#^Patients can have multiple sources of infection, and percentages represent the number of each source out of the BSI group sizes. SOFA: sequential organ failure assessment. Continuous data are displayed as median [interquartile range] and compared using Kruskal–Wallis test; categorical data are displayed as count (percentage) and compared using Fisher’s exact test. | |

Table S9. Targeted analysis of expression of genes in whole blood implicated in syndecan-1 shedding and glycocalyx degradation

| Gene | Category | Direction | rho | p_value | Transcriptome-wide FDR | Target-list FDR |
| --- | --- | --- | --- | --- | --- | --- |
| *MMP8* | MMP-related sheddase / plasma biomarker in current panel | Positive | 0.185 | 0.026 | 0.199 | 0.34 |
| *MMP9* | MMP-related sheddase | Positive | 0.159 | 0.056 | 0.301 | 0.363 |
| *HPSE* | Heparan sulfate / glycocalyx degradation | Positive | 0.055 | 0.51 | 0.804 | 0.789 |
| *ADAMTS1* | Other syndecan sheddase | Positive | 0.027 | 0.746 | 0.918 | 0.856 |
| *ADAMTS4* | Other syndecan sheddase | Positive | 0.011 | 0.898 | 0.968 | 0.898 |
| *HYAL2* | Hyaluronan / glycocalyx degradation | Positive | 0.065 | 0.439 | 0.762 | 0.789 |
| *ADAM17* | ADAM sheddase | Negative | -0.089 | 0.286 | 0.635 | 0.789 |
| *ADAM10* | ADAM sheddase | Negative | -0.056 | 0.506 | 0.802 | 0.789 |
| *MMP2* | MMP-related sheddase | Negative | -0.047 | 0.577 | 0.841 | 0.789 |
| *MMP7* | MMP-related sheddase | Negative | -0.139 | 0.0946 | 0.385 | 0.41 |
| *MMP14* | Membrane-type MMP | Negative | -0.043 | 0.607 | 0.858 | 0.789 |
| *HYAL1* | Hyaluronan / glycocalyx degradation | Negative | -0.075 | 0.372 | 0.714 | 0.789 |
| *F2* | Thrombin precursor / coagulation protease system | Negative | -0.022 | 0.79 | 0.936 | 0.856 |
| *MMP16* | Not available | NA | NA | NA | NA | NA |
| *PLG* | Not available | NA | NA | NA | NA | NA |

Spearman correlations were calculated between plasma syndecan-1 levels and whole-blood gene expression. Target-list FDR was calculated across the available genes in the predefined shedding/glycocalyx gene list. Transcriptome-wide FDR was calculated across all genes in the transcriptomic dataset.

Figure S1: Kaplan-Meier 30-Day Survival Curve Stratified by Syndecan-1 Tertiles


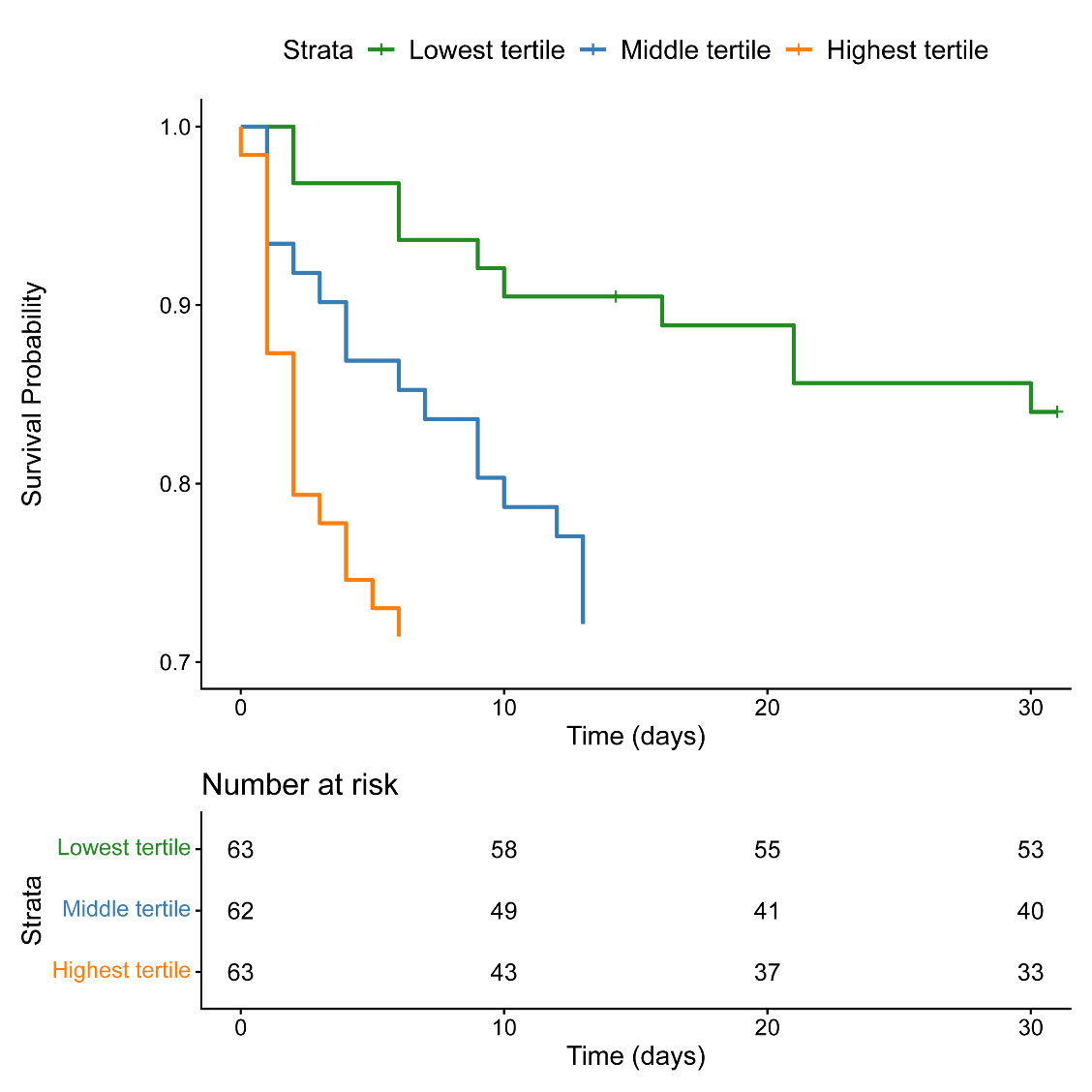


Figure S2. Sensitivity analysis adjusted for plasma creatinine


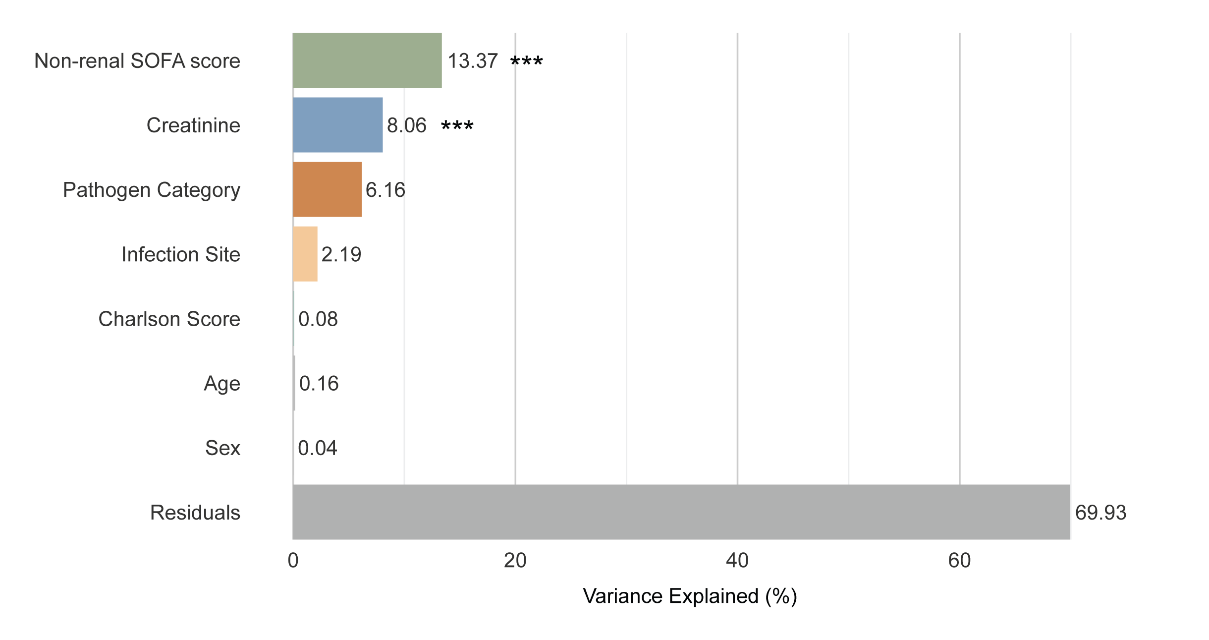


Legend Figure S2: Contribution of renal function, non-renal disease severity and other variables to variation in plasma syndecan-1 levels. Linear regression was performed on log₁₀-transformed syndecan-1 levels. Total SOFA was replaced by non-renal SOFA score, and log₁₀-transformed plasma creatinine was added to the model. Other covariates included pathogen category, infection site, Charlson score, age and sex. Bars show the proportion of total variance in log₁₀-transformed syndecan-1 levels explained by each covariate. Residuals indicate unexplained variance. Colors reflect variable categories. ***: P < 0.001.

Figure S3: PCA of host response pathways across Syndecan-1 tertiles


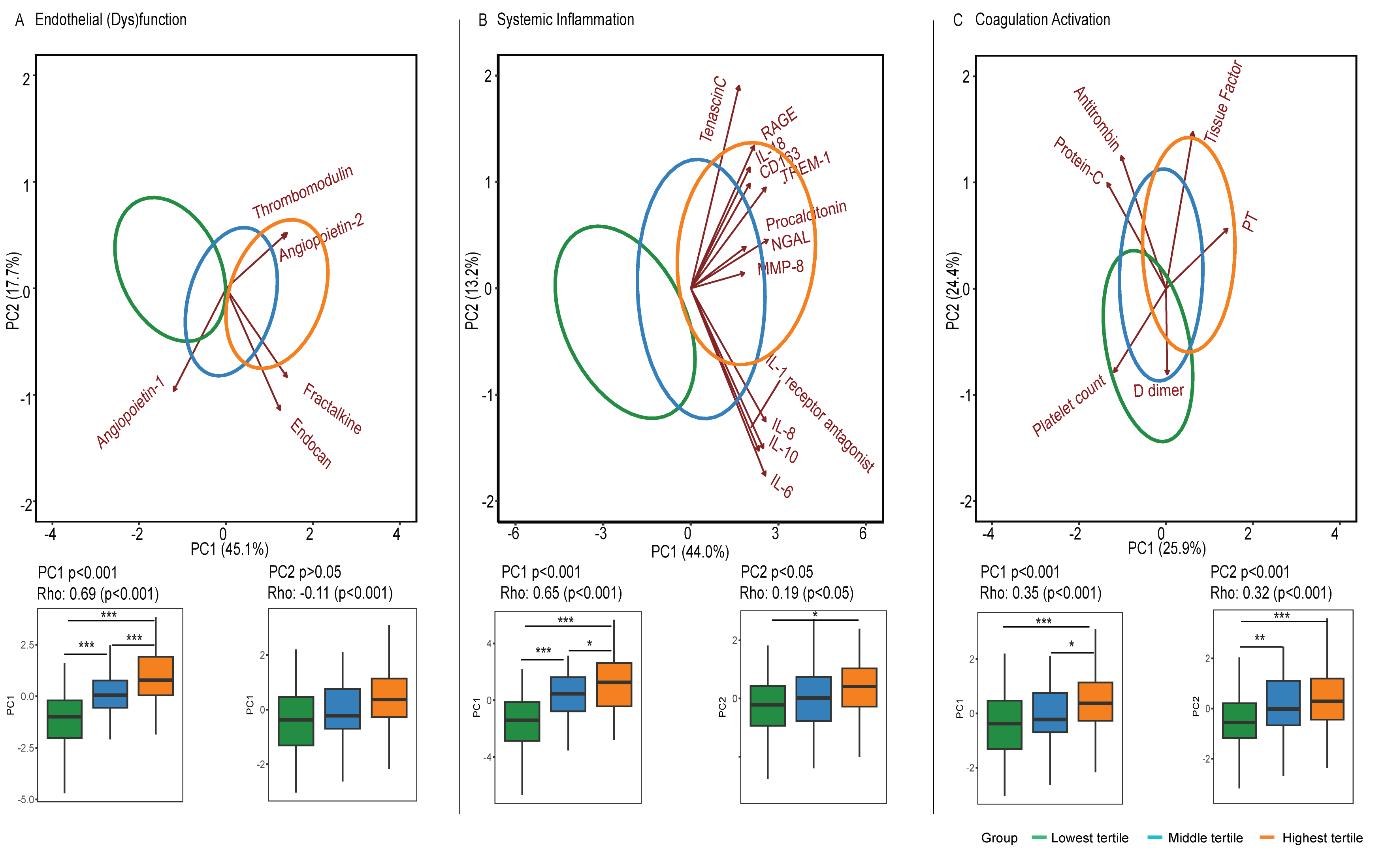


Legend Figure S3: Principal component analysis (PCA) of three host response pathways, grouped by Syndecan-1 levels. shows A) Endothelial cell (dys)function. B) Systemic inflammation. C) Coagulation activation. Colors reflect Syndecan-1 groups, and ellipses mark the 25% confidence area for each group to illustrate how samples are distributed in the PCA space. The x- and y-axes show the first two principal components (PC1 and PC2), along with the percentage of total variance they explain. Arrows indicate the contribution and direction of each biomarker to the PCA. Longer arrows mean a stronger influence on the components. Differences in PCA scores between groups were tested using one-way ANOVA, with post-hoc comparisons based on Tukey’s test. Asterisks mark significant differences (*p < 0.05, **p < 0.01, ***p < 0.001, ****p < 0.0001). Pearson correlations between Syndecan-1 and PCA scores (PC1 or PC2) are also shown. Detailed loading values for each biomarker are available in Supplementary Table S4. For abbreviations see Table S1.

Figure S4 Sensitivity analysis of the coagulation domain principal component analysis in patients in whom all biomarkers were measured (excluding those with imputed antithrombin and protein C levels)


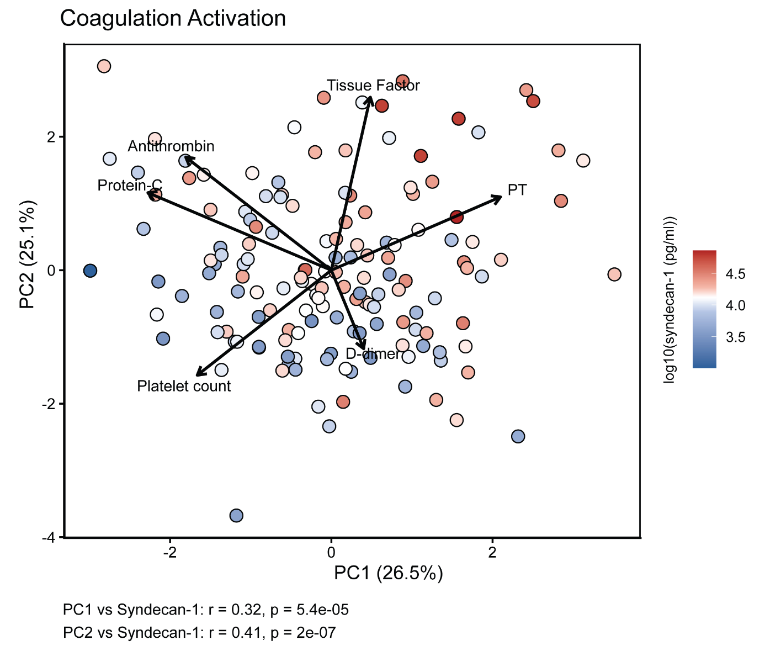


Legend Figure S3: This sensitivity analysis repeated the PCA of coagulation markers using only patients in whom all biomarkers were measured (n =151), i.e., excluding patients with imputed antithrombin and protein C concentrations. Syndecan-1 was included as a continuous variable. Each point shows one patient, colored by log₁₀-transformed syndecan-1 levels. PC1 and PC2 are shown on the x- and y-axes, with the explained variance indicated in parentheses. Black arrows show the loadings of each biomarker, and longer arrows indicate a stronger contribution.

Figure S5: Differentially expressed genes and pathway enrichment results comparing the lowest to the highest syndecan-1 tertile


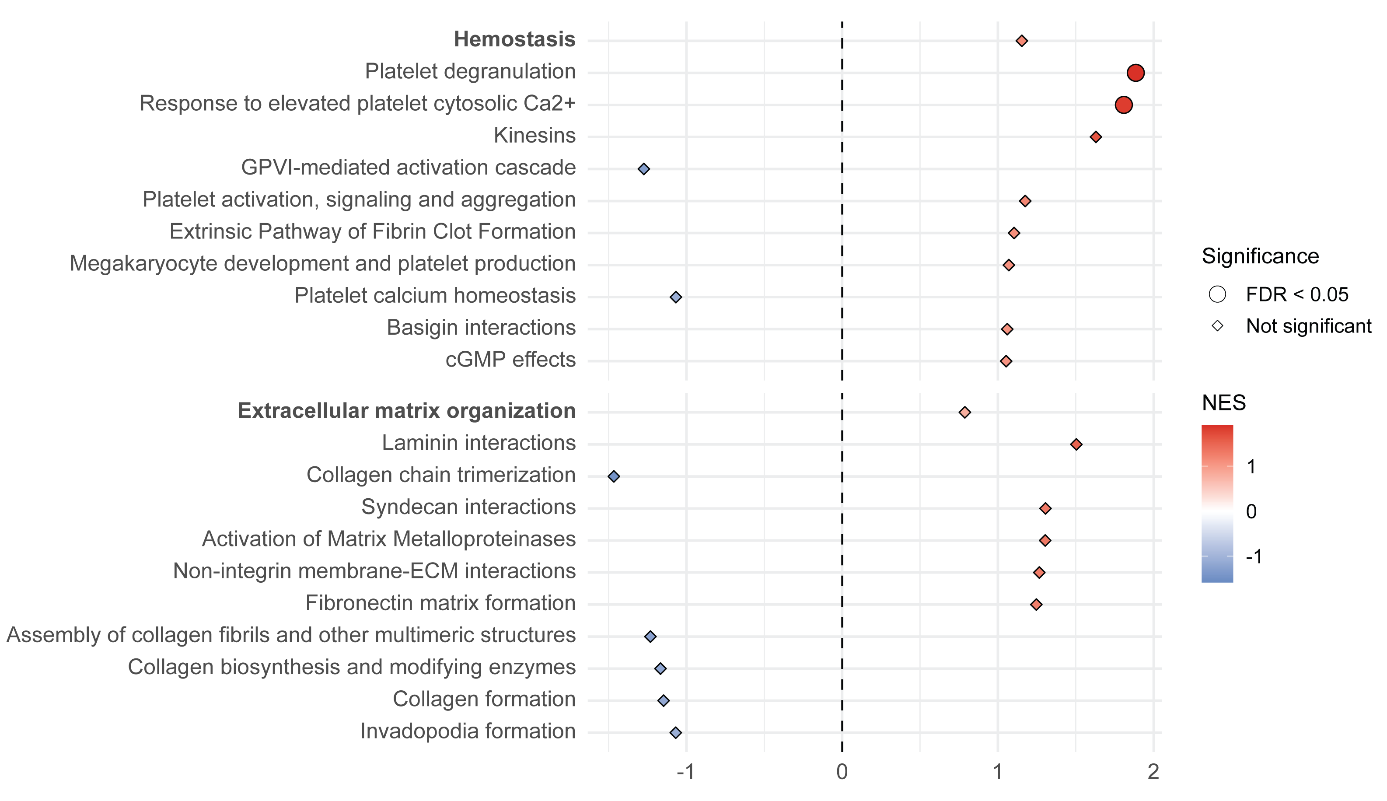


Legend Figure S4: Reactome pathway enrichment results focused on Hemostasis and Extracellular Matrix Organization. Parent pathways are shown in bold; child pathways are in lighter font. The x-axis shows the normalized enrichment score (NES). The dot shape indicates significance (circles for FDR < 0.05, diamonds for not significant), dot size reflects enrichment strength, and color shows direction—red for enrichment in the highest syndecan-1 group, blue for the lowest. The top ten enriched child pathways for each category are shown.
